# Localized cardiolipin synthesis is required for the assembly of MreB during the polarized cell division of *Chlamydia trachomatis*

**DOI:** 10.1371/journal.ppat.1010836

**Published:** 2022-09-12

**Authors:** Scot P. Ouellette, Laura A. Fisher-Marvin, McKenna Harpring, Junghoon Lee, Elizabeth A. Rucks, John V. Cox

**Affiliations:** 1 Department of Pathology and Microbiology, University of Nebraska Medical Center, Nebraska Medical Center, Omaha, Nebraska; 2 Department of Microbiology, Immunology, and Biochemistry, University of Tennessee Health Science Center, Memphis, Tennessee; University of Virginia School of Medicine, UNITED STATES

## Abstract

Pathogenic *Chlamydia* species are coccoid bacteria that use the rod-shape determining protein MreB to direct septal peptidoglycan synthesis during their polarized cell division process. How the site of polarized budding is determined in this bacterium, where contextual features like membrane curvature are seemingly identical, is unclear. We hypothesized that the accumulation of the phospholipid, cardiolipin (CL), in specific regions of the cell membrane induces localized membrane changes that trigger the recruitment of MreB to the site where the bud will arise. To test this, we ectopically expressed cardiolipin synthase (Cls) and observed a polar distribution for this enzyme in *Chlamydia trachomatis*. In early division intermediates, Cls was restricted to the bud site where MreB is localized and peptidoglycan synthesis is initiated. The localization profile of 6xHis tagged Cls (Cls_6xH) throughout division mimicked the distribution of lipids that stain with NAO, a dye that labels CL. Treatment of *Chlamydia* with 3’,6-dinonylneamine (diNN), an antibiotic targeting CL-containing membrane domains, resulted in redistribution of Cls_6xH and NAO-staining phospholipids. In addition, 6xHis tagged MreB localization was altered by diNN treatment, suggesting an upstream regulatory role for CL-containing membranes in directing the assembly of MreB. This hypothesis is consistent with the observation that the clustered localization of Cls_6xH is not dependent upon MreB function or peptidoglycan synthesis. Furthermore, expression of a CL-binding protein at the inner membrane of *C*. *trachomatis* dramatically inhibited bacterial growth supporting the importance of CL in the division process. Our findings implicate a critical role for localized CL synthesis in driving MreB assembly at the bud site during the polarized cell division of *Chlamydia*.

## Author summary

The obligate intracellular human pathogen *Chlamydia trachomatis* is the leading bacterial cause of sexually transmitted infections. *C*. *trachomatis* does not express FtsZ, which is necessary for the highly conserved process of binary fission that most bacteria use to divide. In the absence of FtsZ, *C*. *trachomatis* undergoes a polarized mechanism of cell division that is dependent upon the rod-shape determining protein MreB, which is necessary for septal peptidoglycan synthesis in dividing cells. How MreB is recruited to specific sites in this coccoid organism to initiate polarized division was unclear prior to this study. Here, we show that the polarized synthesis of the phospholipid, cardiolipin, by cardiolipin synthase is necessary for the recruitment of MreB to the site where the nascent daughter cell will form in dividing organisms. Disrupting the localization or function of cardiolipin in *C*. *trachomatis* prevents the assembly of MreB at division sites and inhibits bacterial growth. Our data demonstrate that the polarized synthesis of cardiolipin directs the recruitment of MreB, a key coordinator of division in *Chlamydia*, to the site where the nascent daughter cell will form in dividing cells.

## Introduction

The obligate intracellular bacterium *Chlamydia* undergoes a unique biphasic developmental cycle that alternates between a non-dividing infectious form, the elementary body or EB, and a non-infectious dividing form, the reticulate body or RB [1]. *Chlamydia* species such as *C*. *trachomatis* and *C*. *pneumoniae* are major pathogens of humans causing sexually transmitted infections and community acquired pneumonia, respectively [2–5]. Due to their obligate intracellular nature, an understanding of the underlying mechanisms these bacteria use to accomplish essential processes is lacking. However, the recent development of selected genetic tools for *Chlamydia* has facilitated studies on the basic microbiology of these unique bacteria [6–9].

*Chlamydia* has undergone significant genome reduction in evolving to obligate intracellular dependence [10]. Interestingly, even after genomic reduction, *Chlamydia*, a coccoid bacterium, encodes a number of rod-shape determining proteins including the actin-like protein, MreB [11–14]. One gene that has been lost in these bacteria is *ftsZ*, which encodes the tubulin-like FtsZ protein that orchestrates the cell division process in most bacteria [15]. Whereas most model bacteria divide by FtsZ-dependent binary fission, we have demonstrated that *Chlamydia* undergoes an MreB-dependent polarized cell division process [11,13,16]. This budding-like mechanism of cell division initiates with the synthesis of a patch of peptidoglycan at the pole of *Chlamydia trachomatis* where the budding daughter cell will arise. As the polarized division process proceeds and peptidoglycan deposition continues, the peptidoglycan structure can be visualized as a ring that is retained at the septum that forms between the budding daughter cell and the mother cell [13,17,18]. Over time, the bud is enlarged as the peptidoglycan ring grows until the budding daughter cell is equal in volume to the mother cell. At this point, constriction of the dividing cells occurs with concomitant loss of the peptidoglycan structure. Inhibitor studies have revealed that the activity of MreB, which overlaps the distribution of the septal peptidoglycan ring, is essential for the polarized cell division process of *Chlamydia* [11,13,18]. We have further demonstrated that chlamydial MreB has a unique N-terminal domain, as compared to other bacterial orthologs, that possesses membrane binding properties that may allow it to functionally substitute for the lack of FtsZ in these organisms [13]. However, the means by which MreB is recruited to the site of budding was unclear prior to the current study.

The Gram-negative pathogenic *Chlamydia* species are unusual in lacking a classical peptidoglycan sacculus to define their cell shape [18]. Their cell walls are essentially lipid-based and likely adopt the most thermodynamically favorable shape within an aqueous environment: that of a sphere. This morphology presents unique challenges for context-dependent protein localization as the inner leaflet of the inner membrane displays uniform negative membrane curvature. Given that MreB is the key driver of cell division in these bacteria and that cell division is initiated from one pole of the cell [11,16], the uniform curvature of the inner membrane poses an additional conundrum as MreB is excluded from areas of negative membrane curvature in other bacteria [19–21]. In rod-shaped bacteria like *E*. *coli*, one characteristic of the cell poles is that they are enriched in anionic phospholipids (aPLs) like cardiolipin (CL) [22,23]. CL is particularly suited for such sites as it is a conical phospholipid with a small head group and four acyl chains, and its clustering allows it to spontaneously introduce negative membrane curvature [24].

We hypothesized that localized CL synthesis in *Chlamydia* induces membrane curvature that in turn enables MreB recruitment to initiate cell division. Alternatively, CL may directly recruit chlamydial MreB, perhaps through an interaction with its unique N-terminal domain [13]. The major phospholipids in *C*. *trachomatis* are phosphatidylethanolamine (~45–50%) and phosphatidylcholine (~39%), with other phospholipids including CL comprising approximately 5% or less of the total glycerophospholipid content of chlamydiae [25]. A study from the Rock lab demonstrated that *Chlamydia* synthesizes CL and identified a candidate CL synthase (*cls*) gene, *ct284* [26]. To initiate our studies, we performed a bioinformatic analysis of chlamydial *cls* and monitored its transcription during the developmental cycle to determine that it is expressed during the stage when RBs are undergoing growth and division. Inducible expression of a 6xHis tagged Cls in *C*. *trachomatis* revealed that this enzyme accumulated at the bud site where MreB localizes and peptidoglycan synthesis occurs prior to the initiation of daughter cell outgrowth. As the budding process proceeds, 6xHis tagged Cls (Cls_6xH) is maintained at the leading edge of the daughter cell, while 6xHis tagged MreB (MreB_6xH) is retained at the septum between the daughter and the mother cell. The localization profile of Cls_6xH throughout the division process mimicked the distribution of endogenous lipids that stain with NAO, a dye that labels aPLs, including CL. Treatment of *Chlamydia* with 3’,6-dinonylneamine (diNN), an antibiotic that targets CL-containing membrane domains [27,28], resulted in a redistribution of Cls_6xH and NAO staining aPLs. Importantly, MreB_6xH localization was significantly altered in diNN-treated cells suggesting an upstream regulatory role for CL-containing membranes in directing the assembly of MreB. This hypothesis is consistent with the observation that the clustered localization of Cls_6xH is not dependent upon MreB function or peptidoglycan synthesis. Furthermore, the expression of a mitochondrial derived CL-binding protein [29] at the inner membrane of *C*. *trachomatis* dramatically inhibited bacterial growth supporting the importance of CL in the division process. Our findings implicate polarized CL synthesis as an early event that is critical for the recruitment of MreB to the bud site during the polarized cell division process of *Chlamydia*.

## Results

### The cardiolipin synthase ortholog of *C. trachomatis* localizes to the leading edge of the dividing RB

In 2015, a chlamydial cardiolipin synthase was bioinformatically identified by Yao et al. but not further characterized [26]. This same study identified changes in CL content within infected cell cultures that were consistent with a chlamydial origin, concluding that *Chlamydia* synthesizes its own CL from the phosphatidic acid pool [26]. The gene designation for *cls* in *C*. *trachomatis* is *ct284/ctl0536*. The chlamydial *cls* is conserved in pathogenic *Chlamydia* species (S1A Fig), which lack a peptidoglycan sacculus, and weak homology (<50% similarity) outside of the enzymatic domains was found to orthologous proteins in more distantly related *Chlamydia* genera like *Protochlamydia* [30] as well as other bacteria like *E*. *coli* and *B*. *subtilis* that possess peptidoglycan saccula. As an initial indicator of when Cls functions during the chlamydial developmental cycle [31], we monitored its transcription by RT-qPCR. Transcripts for *cls* were not reliably detected at the 0 and 1h post-infection (hpi) timepoints but increased from 3 to 16hpi before declining (S1B Fig). Peak transcription at 16hpi is consistent with the RB phase of the developmental cycle [12,32], which suggests a potential function for Cls in RB growth and division.

To initiate our studies of the function of Ct284/Cls, we created a transformant of *C*. *trachomatis* L2 carrying a plasmid with an anhydrotetracycline (aTc)-inducible hexahistidine-tagged Cls (Cls_6xH). We then assessed the localization of the Cls_6xH protein in *Chlamydia*. After infecting cells with this transformant, we induced expression and fixed the cells at 10.5hpi, a time when they are undergoing their first division [16]. As we have described previously, the major outer membrane protein (MOMP) is highly polarized to one side of the cell at this stage of the developmental cycle, and the daughter cell arises from the MOMP-enriched pole of the mother cell [16]. Of note, although MOMP is enriched on one side of the bacterium, it is still present at lower levels around the periphery of the mother cell. For this and subsequent experiments, we have defined several intermediates in the chlamydial polarized division process. Pre-division intermediates (“round” cells) have no obvious outgrowth of the budding daughter cell. In “early” division intermediates the daughter cell is <15% of the mother cell volume, in “mid/late” division intermediates the daughter cell is between 15–80% of the mother cell volume, and in the “two-cell” stage of division the daughter cell is >80% of the mother cell volume.

**Fig 1:**
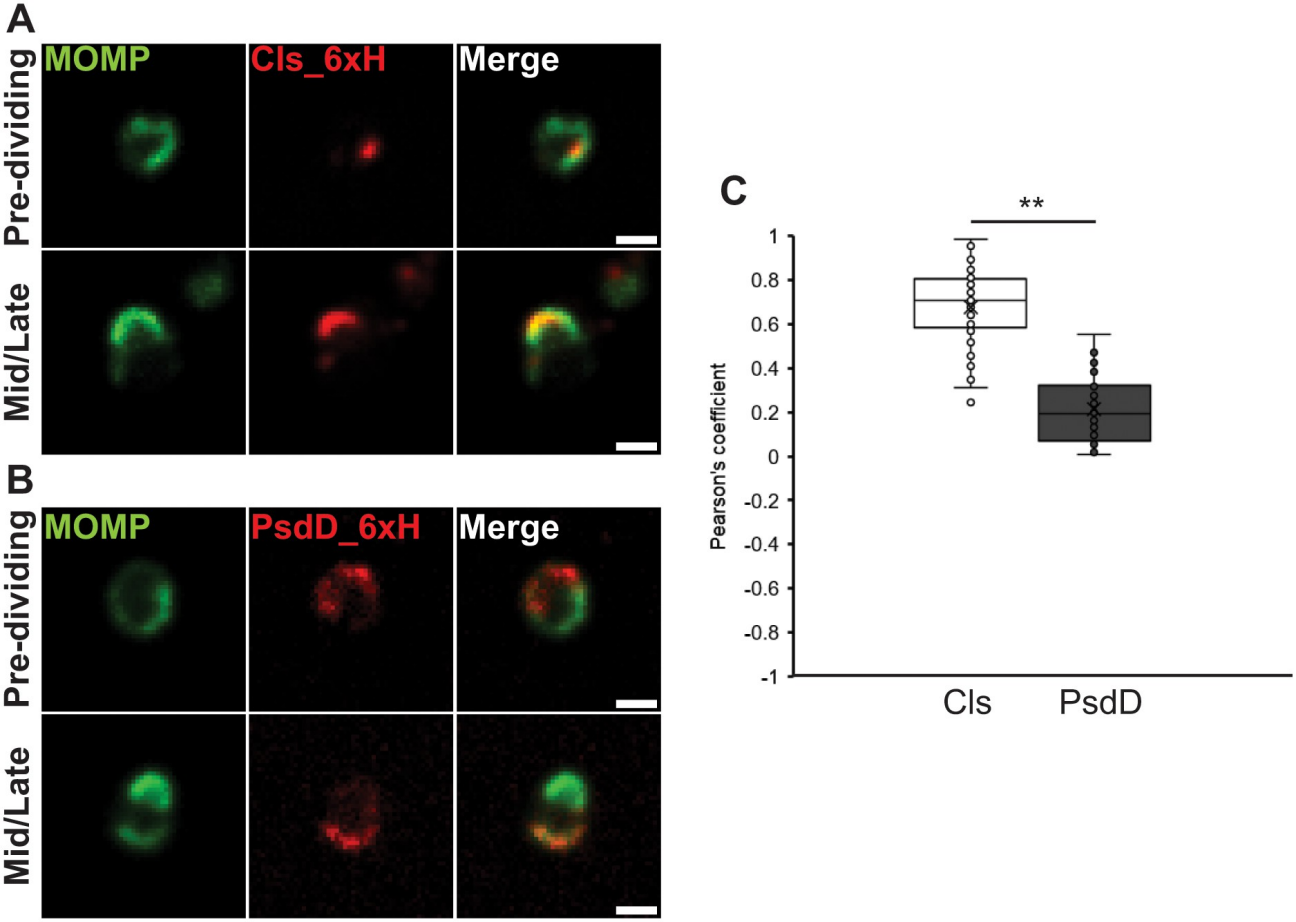
Localization of Cls_6xH and PsdD_6xH during various stages of the initial RB division. HeLa cells were infected with *C*. *trachomatis* L2 transformants carrying plasmids encoding inducible constructs for (A) Cls_6xH or (B) PsdD_6xH. Expression of the construct was induced at 4hpi with 10nM anhydrotetracycline (aTc). At 10.5hpi, cells were fixed and processed for immunofluorescence analysis as described in Materials and Methods. Shown are representative images for each in a pre-dividing cell (Round) and in a Mid/Late division intermediate. Note the accumulation of Cls_6xH at the leading edge of the budding daughter cell whereas PsdD_6xH primarily accumulates at the opposite side of the dividing cell in the mother cell membrane. Images were acquired on a Nikon Ti2 spinning disc confocal microscope using a 60X lens objective. Images are representative of at least three independent experiments. Scalebar = 2 μm. (C) Quantification of colocalization of Cls_6xH or PsdD_6xH with MOMP as measured using the JACoP plug-in of FIJI. Pearson’s coefficient for 50 individual cells for each transformant is graphed. ** = p<0.001.

Cls_6xH accumulated at the MOMP-enriched side of the mother cell in round cells, and it remained at the leading edge of the budding daughter cell after division started in mid/late intermediates where it overlapped the distribution of MOMP (Fig 1A). To determine whether this was a general feature of phospholipid synthases, we also monitored the localization of PsdD_6xH, which is an annotated phosphatidylethanolamine synthase. In contrast to Cls_6xH, PsdD_6xH localized on the opposite side from the MOMP-enriched pole of the cell both before and after division initiated (Fig 1B). Quantification of colocalization between MOMP and Cls_6xH or PsdD_6xH from 50 individual cells for each strain revealed that Cls_6xH demonstrated a significant colocalization with MOMP (Pearson coefficient of ~0.7) whereas PsdD_6xH exhibited minimal colocalization with MOMP (Pearson coefficient of ~0.2) (Fig 1C). Finally, we compared the localization pattern of these proteins to MreB_6xH, a characterized division protein in *Chlamydia* that substitutes for FtsZ [11,14]. Prior to the initiation of division, MreB_6xH, like Cls_6xH, is clustered at a site on the MOMP-enriched side of the mother cell. However, at the onset of daughter cell growth, MreB_6xH is retained at the septum where it forms a ring structure beneath the growing bud (S2 Fig) [13]. These data demonstrate that Cls_6xH and MreB_6xH both accumulate in polar clusters prior to the initiation of daughter cell growth. However, as the budding process initiates, Cls_6xH is maintained at the leading edge of the budding daughter cell, while MreB_6xH is retained at the septum between the daughter and the mother cell.

To investigate the potential function of Cls at later stages of the developmental cycle, cells were infected with the Cls_6xH transformant, the expression of the fusion was induced or not at 8h post-infection (hpi) with 5nM aTc, and the cells were fixed and processed for immunofluorescence analysis (IFA) at 24hpi. The expression of Cls_6xH had no obvious effect on inclusion size, and the protein exhibited a polar pattern of localization in individual cells within the inclusion (S3A Fig). Overexpression of Cls_6xH did result in a statistically significant effect on the growth of the organism as assessed by inclusion forming unit assay (a chlamydial equivalent to colony forming unit assay). After induction of Cls_6xH with 5nM aTc, chlamydial IFUs were ~23% (p<0.001) of the uninduced, untreated control (S3B Fig). However, overall DNA replication, as measured by the levels of genomic DNA over time, after induction of Cls_6xH was unchanged compared to the uninduced control (S3C Fig). These data suggest that overexpression of Cls_6xH may slightly delay overall developmental cycle progression while having a limited impact on total organism number.

**Fig 2:**
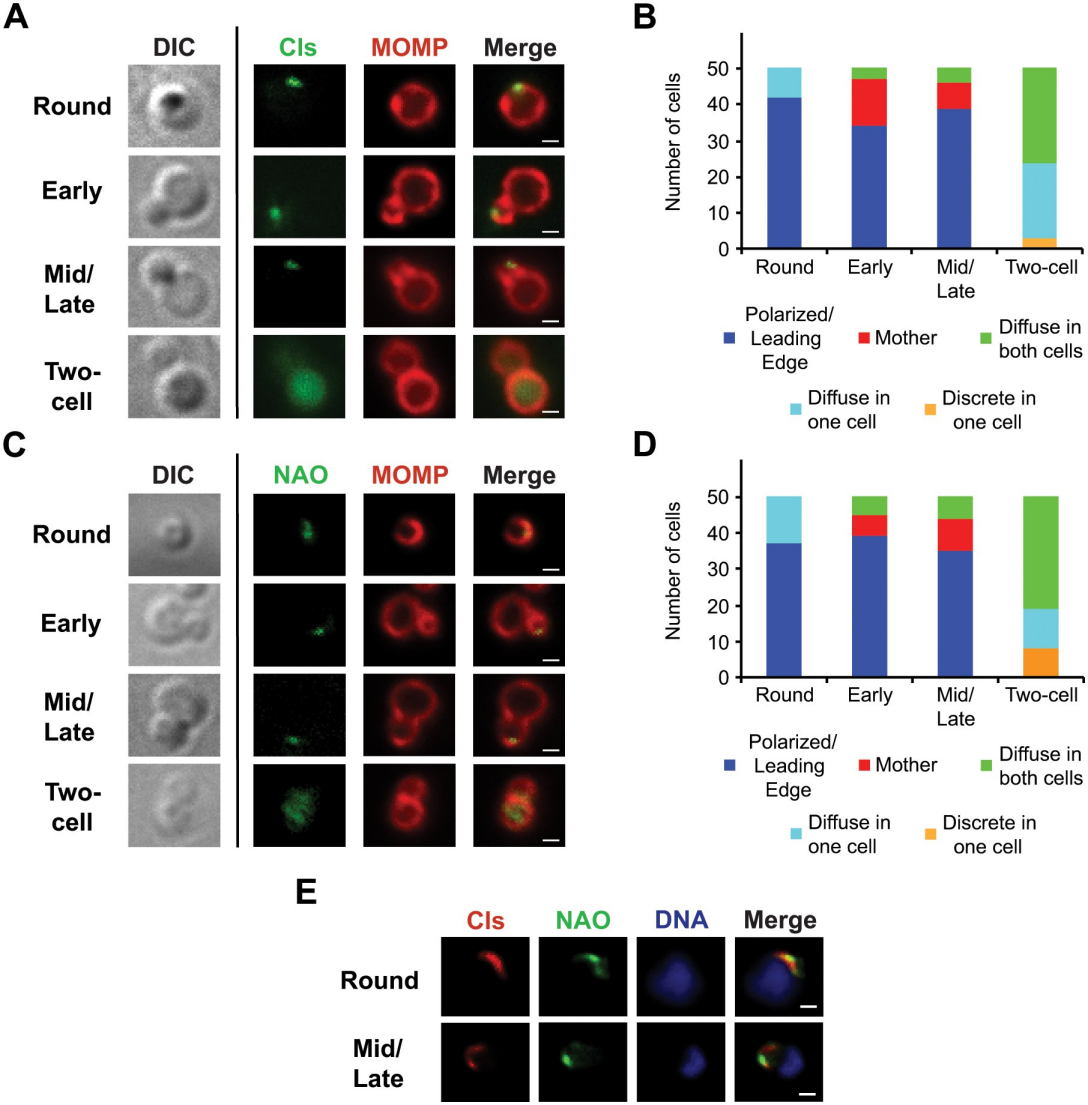
Localization of Cls_6xH and anionic phospholipids (aPLs) in dividing *Chlamydia*. HeLa cells were infected with the Cls_6xH transformant, and expression of the construct was induced at 20hpi with 10nM aTc. The infected cells were lysed at 22hpi as described in Materials and Methods and the distribution of (A and C) MOMP, (A) Cls_6xH (labeled Cls), or (C) aPLs (stained with 250nM NAO for 1 hour) within chlamydial cells in the lysate was assessed. Representative images illustrating the localization profiles for each marker in Round cells (pre-division intermediates), and in the Early, Mid/Late, and Two-cell stages of the polarized division process are shown. Differential interference contrast (DIC) images are shown for each of the division intermediates in panels A and C. The distribution of (B) Cls_6xH and (D) NAO-stained aPLs was evaluated in 50 individual cells from the indicated stages of division. Localization profiles for Cls_6xH and NAO-staining phospholipids were categorized into polarized/leading edge of the budding daughter cell, mother, diffuse in one cell, diffuse in both cells, and discrete in one cell. These data were statistically analyzed using a chi-squared test, and there was no statistical difference between Cls_6xH and NAO localization profiles at any stage of division. (E) Co-localization of Cls_6xH (labeled Cls) with NAO-stained aPLs. The expression of Cls_6xH was induced at 20hpi with 10nM aTc and the infected cells were lysed at 22hpi as described above. The distribution of Cls_6xH, aPLs (using NAO), and DNA (using Hoechst 33342) in Round and Mid/Late stage division intermediates present in the lysate are shown. Images in A, C, and E were acquired with a Zeiss AxioImager2 microscope equipped with a 100x oil immersion PlanApochromat lens. Scalebar = 2 μm.

Since it was difficult to assess the stage of division of cells within the inclusion at the 24h time point, we induced the expression of Cls_6xH with aTc at 20hpi, lysed the infected HeLa cells at 22hpi and imaged individual cells in the lysate. The induction period was shorter for these experiments, so we used a higher concentration of aTc (10nM) to ensure a detectable level of Cls_6xH expression. To determine the effect of this higher level of inducer on chlamydial growth, we measured IFUs at 24hpi after inducing Cls_6xH expression with 10nM aTc at 16hpi (8h total). Under these conditions, we observed ~35% reduction in IFUs that again may be due to a delay in developmental cycle progression (S3D Fig). The morphology of *Chlamydia*, which were induced with 10nM aTc for 2 hrs and prepared from lysates of infected HeLa cells, clearly illustrates the various stages of the polarized division process (Fig 2A). Quantification of the division intermediates present in lysates from chlamydiae induced or not to express Cls_6xH also revealed that Cls_6xH induction did not affect the profile of division intermediates present in infected cells at 22hpi (S3E Fig). Immunofluorescent staining of the induced cells revealed that Cls_6xH localization in dividing RBs at this stage of the developmental cycle was very similar to that observed in cells undergoing their first division (Fig 1A). Cls_6xH accumulated in a polar cluster in round cells and at the leading edge of budding daughter cells in early and mid/late budding intermediates (Fig 2A). At the two-cell stage, the localization of Cls_6xH became more diffuse within the daughter and mother cells (Fig 2A). Quantification of this analysis revealed the observed localization profiles of Cls_6xH at the various stages of polarized budding (Fig 2B).

This approach also enabled us to visualize the distribution of anionic phospholipids (aPLs) in bacteria during the polarized division process. Nonyl acridine orange (NAO) binds the aPLs, CL and phosphatidylglycerol, and this dye has been used to visualize the distribution of these lipids in other bacteria [22,23]. Attempts to use NAO to determine whether the polar Cls_6xH staining observed in *Chlamydia* at 24hpi (S3A Fig) corresponded to regions of the chlamydial membrane enriched in these aPLs were unsuccessful because of the high levels of background staining observed in infected cells stained with this dye. As an alternative approach, we induced the expression of Cls_6xH and prepared a lysate as described above. When the *Chlamydia* in the lysate were stained with NAO for 1 hour, the localization profile of NAO-staining aPLs in cells undergoing division (Fig 2C) was very similar to the distribution of Cls_6xH (Fig 2A). aPLs were primarily in a polar cluster in round cells and were restricted to the leading edge of budding daughter cells in early and mid/late division intermediates (Fig 2C and 2D). At the two-cell stage, the NAO staining became more diffuse, similar to what was observed for Cls_6xH (Fig 2). Although aPLs were not reproducibly detected when uninduced *Chlamydia* were incubated with NAO for 1 hour, longer incubation times with the dye revealed that endogenous aPLs in the uninduced Cls_6xH transformant (S4A and S4B Fig) and in wild-type *Chlamydia trachomatis* serovar L2 (S4C and S4D Fig) exhibited localization profiles similar to the NAO staining observed in cells where Cls_6xH expression was induced. While NAO staining profiles were very similar in the uninduced Cls_6xH transformant and in wild-type *Chlamydia trachomatis* serovar L2, an additional category of NAO staining (diffuse in one cell) was detected in early and mid/late division intermediates in wild-type *Chlamydia trachomatis* that was not detected in the uninduced Cls_6xH transformant (S4 Fig). Whether this difference is due to slight leakiness in the expression of Cls_6xH that affects the distribution of aPLs in the transformed cells is unclear at this time. Finally, the images in Fig 2E illustrate that the localization profile of Cls_6xH overlapped the distribution of NAO-staining phospholipids in induced cells undergoing division. These data strongly suggest that the expression of Cls_6xH induces the accumulation of aPLs at the leading edge of budding daughter cells during polarized division, and the distribution of aPLs following Cls_6xH induction mimicked the distribution profile of endogenous aPLs.

### The transmembrane domain of Ct284/Cls is necessary for its restricted localization

Interestingly, our bioinformatics analyses revealed that the transmembrane (TM) domain region at the N-terminus of the chlamydial Cls is notably shorter than the TM domains of *E*. *coli* or *B*. *subtilis* Cls orthologs. Bacterial cardiolipin synthases typically possess two TM domains such that the enzymatic domain is oriented towards the cytosol [33,34]. TM prediction programs confirmed that the chlamydial Cls possesses only a single predicted TM domain (boxed in green in S1A Fig).

**Fig 3:**
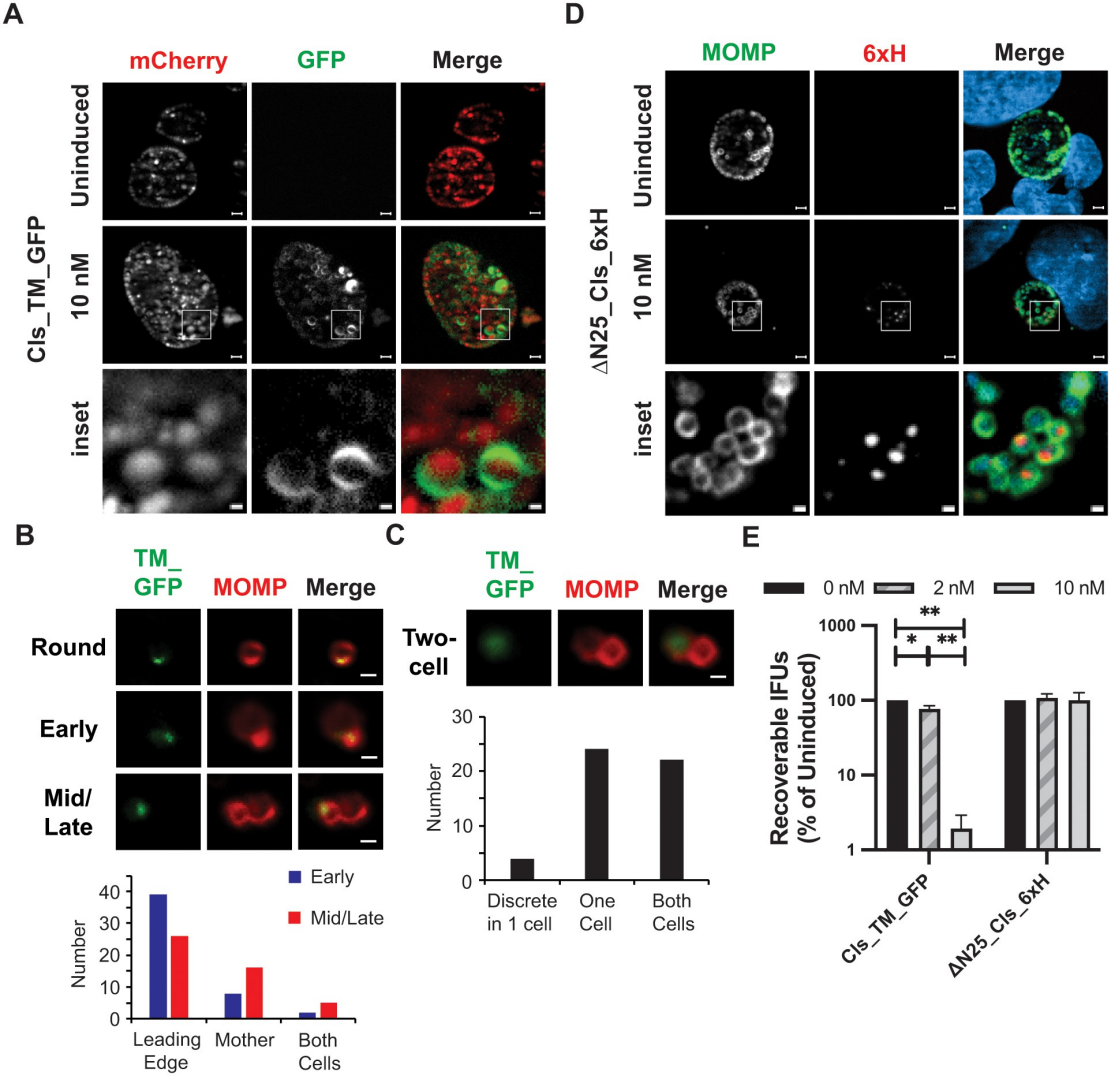
Functional evaluation of the Cls transmembrane (TM) domain in directing protein localization. (A) *C*. *trachomatis* L2 was transformed with an anhydrotetracycline (aTc) inducible vector encoding the Cls TM domain (first 25 residues) fused to GFP; mCherry encoded on the plasmid backbone is constitutively expressed. Cells were infected with this strain, and expression of Cls_TM_GFP was induced or not at 15hpi with 10nM aTc. Localization of the marker and mCherry in live-cell images acquired at 19hpi is shown. (B) The localization of Cls_TM_GFP at Round, Early, and Mid/Late stages of division or (C) the two-cell stage of division was imaged and quantified from transformants induced in axenic media. For (B) Cls_TM_GFP was localized to a polar cluster in round cells and primarily at the leading edge of the budding daughter cells, whereas for (C) it was localized as a discrete patch in one cell or diffuse in one or both cells. At least 50 cells were counted for each stage of division. Images in B and C were acquired with a Zeiss AxioImager2 microscope equipped with a 100x oil immersion PlanApochromat lens. Scalebar in panels B and C = 2 μm. (D) Cells were infected with *C*. *trachomatis* L2 transformed with an aTc-inducible vector encoding Cls_6xH lacking the TM domain (i.e. starting at residue 26). Expression of the construct was induced or not at 4hpi, and samples were fixed and processed at 24hpi as described in the legend to S3 Fig. For (A) and (D) the boxed region within each image is shown below as an enlarged inset. Note the discrete localization pattern to one side of the bacterial membrane in (A) but not in (D). Images in A and D were acquired on a Zeiss AxioImager.Z2 equipped with an Apotome2 using a 100X lens objective. Images are representative of at least three independent experiments. Scalebar of full inclusion images in A and D = 2 μm; Scalebar of inset = 0.5 μm (E) Cells were infected with the indicated transformants and processed as described in the legend to S3 Fig to quantify IFU production during the primary infection. For each transformant, the uninduced values were arbitrarily set to 100%, and the effect of overexpression induced by 2nM or 10nM aTc added at 4hpi is expressed as a percentage of the uninduced control. Data are the average of three independent experiments performed in triplicate. * = p<0.05; ** = p<0.001.

To determine whether the TM domain of Cls was sufficient to direct its restricted membrane localization, we created a chlamydial transformation plasmid encoding an aTc-inducible construct where the chlamydial Cls TM domain was fused to GFP (Cls_TM_GFP). This plasmid also constitutively expresses mCherry. Transformants of *C*. *trachomatis* serovar L2 were obtained, and the fusion protein was expressed after infecting a monolayer of cells. As seen in Fig 3A, the GFP signal was detected and localized to discrete sites on the RB membrane in live-cell images. These data indicate that the TM domain of Cls is sufficient to direct its clustered localization. Further, given that the GFP signal was detected in live cells, these data suggest that the TM domain of Cls orients the protein such that the enzymatic domain is cytosolic, consistent with other Cls orthologs [33,34].

Since it was again difficult to assess the distribution of Cls_TM_GFP during specific stages of division when infected cells were analyzed (Fig 3A), we lysed infected cells at 22hpi and induced the expression of Cls_TM_GFP in chlamydial cells in the lysate by adding aTc to the cells that were incubated in axenic media (Optimem supplemented with 1mM glucose-6-phosphate and 1mM glutamine [35]) for 1 hour. IFA analyses of these cells and quantification of the localization patterns revealed that Cls_TM_GFP exhibited a localization profile indistinguishable from Cls_6xH, as it accumulated in a polar cluster in rounds cells and at the leading edge of budding daughter cells at early and mid/late stages of division (Fig 3B). At the two-cell stage, the localization of Cls_TM_GFP became more diffuse within the mother cell or both cells (Fig 3C).

Given the discrete localization of GFP when fused to the Cls TM domain and the discrete localization of full-length Cls_6xH (Figs 1 and 2), we next determined if the TM domain was critical for the clustered localization of Cls. To test this, we created an N-terminal truncation of Cls_6xH lacking its TM domain (Δ25Cls_6xH) and expressed this in *Chlamydia*. As seen in Fig 3D, Δ25Cls_6xH localized to the cytosol of the bacteria. We conclude from these data that the TM domain of the chlamydial Cls directs its restricted localization to specific regions of the inner membrane.

Finally, we determined the impact of overexpression of the Cls_TM_GFP and Δ25Cls_6xH constructs on chlamydial growth using the IFU assay (Fig 3E). Infected cells were treated with aTc or not at 4hpi using two different concentrations of inducer. We did not measure any growth defects from overexpressing the Δ25Cls_6xH construct but did note that extended overexpression of Cls_TM_GFP with 10nM, but not 2nM, aTc resulted in abnormally enlarged bacteria (S5 Fig) and fewer infectious progeny (Fig 3E), suggesting that accumulation of the Cls_TM domain in the inner membrane may alter membrane organization and disrupt normal cell division.

### Cls_6xH localization is dependent upon its interaction with CL-containing membrane microdomains

3’,6-dinonylneamine (diNN) is an amphiphilic aminoglycoside that interacts with aPLs [27,28]. Treatment of *P*. *aeruginosa* with this drug induced the redistribution of NAO-staining phospholipids from the poles to the sidewall of the cell and affected the permeability and morphology of this organism [36]. Concentrations of diNN that were lower than that required to alter lipid localization and cell growth of *P*. *aeruginosa* were toxic to HeLa cells, so the effect of the drug on Cls_6xH localization could not be monitored by treating *Chlamydia*-infected cells. To circumvent this, we monitored the effect of diNN on *Chlamydia* transformants in lysates prepared from infected cells at 22hpi.

**Fig 4:**
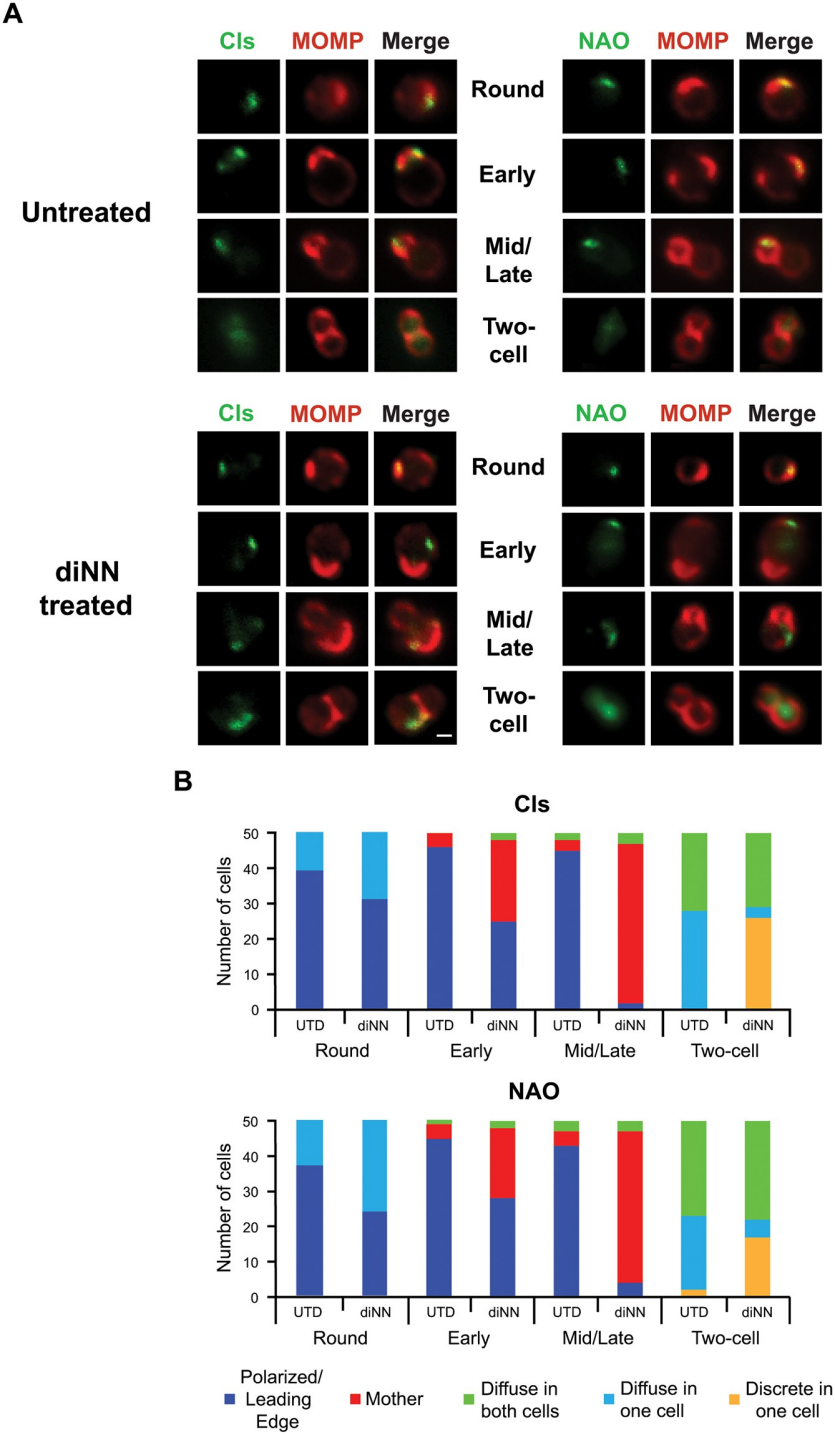
Effect of the CL-targeting antibiotic 3’,6-dinonylneamine (diNN) on the localization of Cls_6xH and aPLs at different stages of the division process. HeLa cells were infected with the Cls_6xH transformant, and at 22hpi, infected cells were lysed and Cls_6xH expression was induced by incubating the cells in the lysate for 1.5hrs. in axenic media containing 10nM aTc in the presence or absence of 5 μM diNN. Cells were then fixed and the distribution of MOMP, Cls_6xH (labeled Cls), or NAO in chlamydial cells was assessed. (A) Representative images of the indicated markers in Untreated or diNN treated bacteria at the indicated stages of cell division. Images were acquired with a Zeiss AxioImager2 microscope equipped with a 100x oil immersion PlanApochromat lens and are representative of two independent experiments. Scalebar = 2 μm (B) 50 individual cells from the indicated stages of division from untreated (UTD) and diNN-treated cells were assessed for their Cls_6xH and NAO localization profiles. Localization profiles were categorized into polarize/leading edge of daughter cell, mother, diffuse in one cell, diffuse in both cells, or discrete in one cell. The differences in localization of Cls_6xH and NAO between treatment conditions at each stage of division were statistically analyzed using a chi-squared test. This revealed that the changes induced by diNN treatment were statistically significant (p<0.001) for each division intermediate compared to untreated (UTD) controls. There was no statistical difference between Cls_6xH and NAO localization profiles for any condition tested.

When the expression of Cls_6xH was induced in the transformants incubated in axenic media containing aTc for 1.5hrs, Cls_6xH and NAO-staining phospholipids were primarily restricted to the leading edge of budding daughter cells in polarized division intermediates (Fig 4A), very similar to the result obtained when the expression of the fusion protein was induced during infection (Fig 2). Quantification of the staining profiles observed in cells incubated in axenic media (Fig 4B) revealed that both Cls_6xH and NAO were in a polar cluster in round cells and present at the leading edge of the daughter cell in most cells during early and mid/late stages of division. The other observed localization profiles primarily reflected the distribution of Cls_6xH and NAO staining aPLs during the two-cell stage prior to cell constriction and separation. To assess the effect of diNN on Cls_6xH and aPL localization, cells were incubated in axenic media containing aTc and 5μM diNN for 1.5hrs. In most of the cells, this treatment resulted in a dramatic and statistically significant redistribution of both Cls_6xH and NAO staining to a discrete region in the mother cell membrane (Fig 4A and 4B). In a subset of the early division intermediates treated with diNN, the distribution of Cls_6xH and NAO-staining phospholipids was similar to that seen in untreated controls. Importantly, there was no statistically significant difference between the localization profiles observed for Cls_6xH and NAO staining aPLs at the different stages of division and between treatments. The molecular basis for the differential effect of diNN on the localization of Cls_6xH and NAO staining aPLs in cells at varying stages of division is unclear at this time.

Additional studies revealed that the response of Cls_TM_GFP to diNN treatment was virtually identical to Cls_6xH, as Cls_TM_GFP redistributed to a discrete region in the mother cell membrane when the drug was included in axenic media during induction (S6A and S6B Fig). Like Cls_6xH, Cls_TM_GFP was retained in the leading edge of budding daughter cells in a subset of the early division intermediates treated with the drug (S6A and S6B Fig). Taken together these results indicate that the TM domain is sufficient to direct Cls to the leading edge of budding cells, and its restricted localization is dependent upon its association with CL-rich membrane microdomains.

To determine whether diNN has a general effect on the localization of phospholipid synthases in *Chlamydia*, we characterized the distribution of PsdD_6xH induced in axenic culture in the absence and presence of diNN. Similar to the results obtained at 10.5hpi (Fig 1B), PsdD_6xH primarily accumulated in the mother cells in polarized division intermediates, and diNN had no apparent effect on its localization profile (S7 Fig). These results strongly suggest that the accumulation of Cls_6xH at the leading edge of budding cells is at least in part dependent upon its interaction with aPLs via its transmembrane domain, and they illustrate a clear difference in the role of aPLs in regulating the localization of Cls_6xH and PsdD_6xH.

### Ectopic expression of a CL/phosphatidic acid binding protein in *Chlamydia* severely disrupts organism growth and morphology

**Fig 5:**
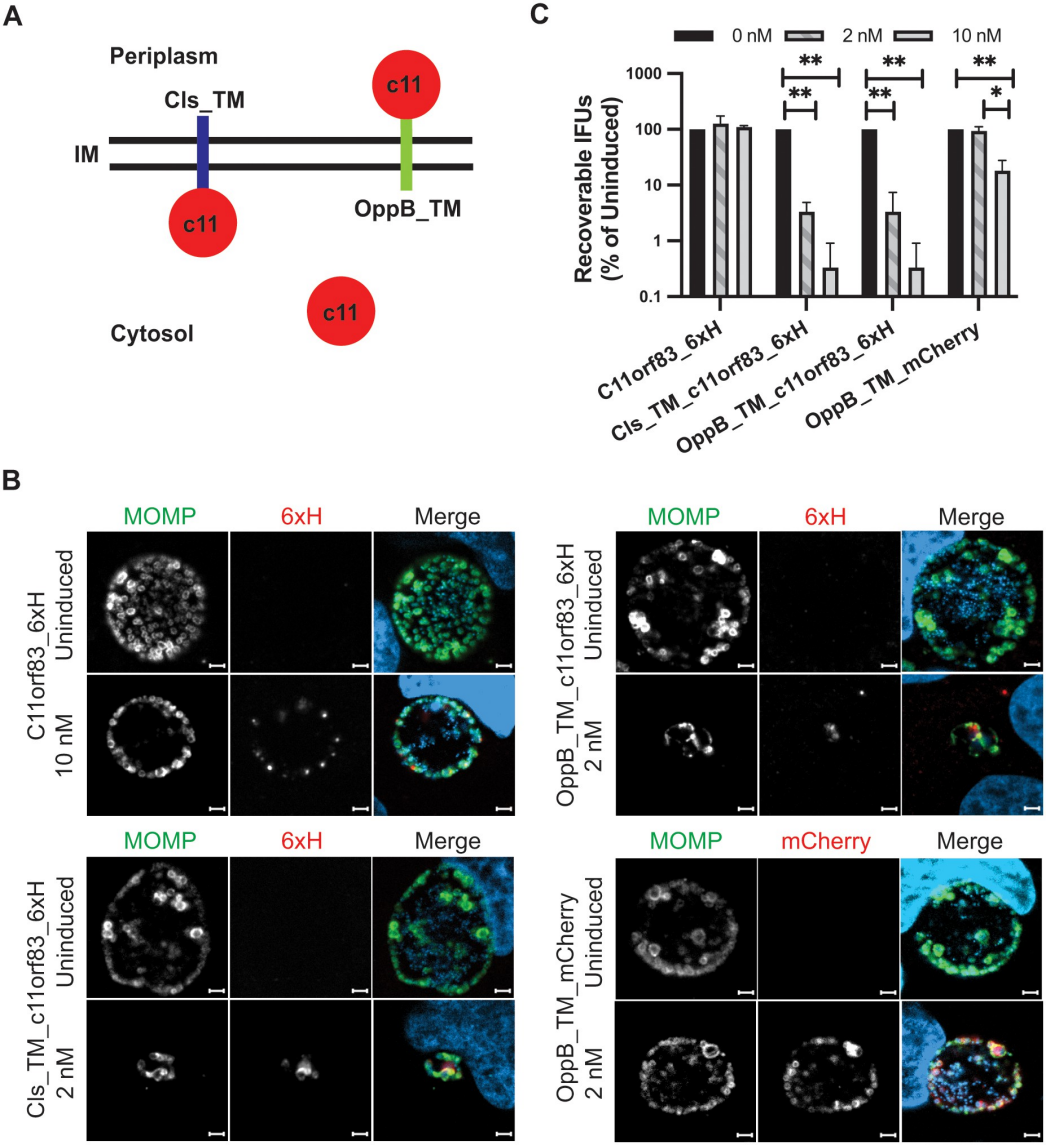
Effect of overexpressing the cardiolipin binding protein, C11orf83, on chlamydial growth and morphology. (A) Schematic of the expected localization of each C11orf83 (c11) construct when expressed in chlamydiae. IM = inner membrane; Cls_TM = first 25 residues of Cls fused to c11; OppB_TM = first TM domain of *E*. *coli* OppB fused to c11 (or mCherry). (B) Cells were infected with *C*. *trachomatis* L2 transformants carrying anhydrotetracycline (aTc) inducible plasmids and encoding the indicated constructs. Expression of each was induced or not at 8hpi with 2 or 10nM aTc as indicated, and cells were fixed and processed for immunofluorescence at 24hpi as described in the legend to S3 Fig. Images were acquired on a Zeiss AxioImager.Z2 equipped with an Apotome2 using a 100X lens objective. Images are representative of at least three independent experiments. Scalebar = 2 μm (C) Cells were infected with the indicated transformants and processed as described in the legend to S3 Fig to quantify IFU production during the primary infection. For each transformant, the uninduced values were arbitrarily set to 100%, and the effect of overexpression at either 2nM or 10nM aTc when added at 4hpi is expressed as a percentage of the uninduced control. Samples were collected at 24hpi. Data are the average of three independent experiments performed in triplicate. * = p<0.05; ** = p<0.001.

As we could not treat infected cells with diNN to determine effects of disrupting CL containing membrane microdomains on chlamydial growth and morphology, we overexpressed a CL-binding protein in *Chlamydia* with the hypothesis that this would interfere with normal CL function. In 2015, a small mitochondrial protein, C11orf83, was characterized as a CL-binding protein with an N-terminal membrane anchor [29]. Although C11orf83 also has affinity for phosphatidic acid (PA) and sulfatide, there is no evidence that *Chlamydia* has sulfatide in its membranes [25,26], and PA is typically short-lived (in *E*. *coli*), and rapidly incorporated into other phospholipids [37,38]. We constructed several codon-optimized C11orf83 variants for aTc-inducible expression in *Chlamydia*: C11orf83_6xH, Cls_TM_c11orf83_6xH (appending the Cls TM domain to the full length C11orf83), and OppB_TM_c11orf83_6xH (replacing the mitochondrial TM domain with a TM domain from *E*. *coli* OppB to target C11orf83_6xH to the periplasm). We previously validated the use of the *E*. *coli* OppB first TM domain to target a protein into the periplasm [39]. The predicted localization of these constructs is depicted in Fig 5A.

We infected cells with these transformants and induced the expression of the fusion proteins at 8hpi with aTc, and then fixed and processed the cells for IFA at 24hpi. Although full-length C11orf83_6xH appeared polar in a subset of the cells, it primarily accumulated in the cytosol (Fig 5B), indicating that the mitochondrial TM domain was not capable of inserting into the chlamydial inner membrane. No obvious effects were noted on inclusion size or bacterial morphology when overexpressing this construct with 10nM aTc. In contrast, when this protein was tethered to the cytosolic facing side of the inner membrane with the Cls_TM domain, a dramatic reduction in chlamydial inclusion size was observed even when induced with a low concentration of aTc (2nM), and this correlated with reduced bacterial numbers and abnormal bacterial cell shape. Note that this effect was not observed when the Cls_TM_GFP expression was induced with 2nM aTc (Fig 3), indicating a specific effect of the C11orf83 at the membrane. Similar effects on inclusion size, bacterial numbers, and cell shape were observed when C11orf83_6xH was expressed in the periplasm and tethered to the outer leaflet of the inner membrane using the first TM domain from OppB of *E*. *coli* (Fig 5B). To ensure that the different effects of the C11orf83 variant constructs on chlamydial growth were not due to differences in the expression levels of the proteins, we collected protein lysates from cells infected with the three strains that were induced with 0, 2, or 10nM aTc. Given the significant impacts on chlamydial inclusion size when C11orf83 was expressed at the membrane specifically, we added aTc at 16hpi and collected lysates at 24hpi to ensure adequate chlamydial biomass for western blotting analysis. These experiments revealed that aTc induction resulted in a comparable level of expression for each of the C11orf83_6xH constructs. While the constructs were detected at similar levels, a significant effect on chlamydial inclusion size was only observed when C11orf83_6xH was tethered to the membrane by the Cls or OppB TM domain (S8 Fig). As a control for overexpressing a protein in the periplasm, we expressed mCherry in the periplasm using the same OppB TM domain and observed its effects. In contrast to OppB_TM_c11orf83_6xH where induction with as little as 2nM aTc was sufficient to cause a dramatic decrease in inclusion size, the expression of OppB_TM_mCherry in the periplasm had minimal effect on inclusion size (Fig 5B).

To quantify the growth effects related to overexpression of the C11orf83 constructs or OppB_TM_mCherry, we performed an IFU assay to measure EB production from the various induced and uninduced cultures. As seen in Fig 5C, and consistent with our immunofluorescence analysis, overexpression of mCherry in the periplasm or C11orf83_6xH in the cytosol had a modest effect or no effect on chlamydial growth. In contrast, when C11orf83_6xH was tethered to either face of the inner membrane, there was a significant and dramatic decrease in IFUs. Collectively, these data indicate that expression of the CL/PA-binding protein in the inner or outer leaflet of the chlamydial inner membrane is sufficient to disrupt chlamydial cell division.

### Cls_6xH localization is not altered by MreB or peptidoglycan synthesis inhibitors

Given the hypothesized role of Cls in cell division, we attempted to determine interactions between it and known cell division proteins using a bacterial adenylate cyclase-based two hybrid system (BACTH) [39,40]. However, we were unable to detect interactions between Cls and any chlamydial cell division (e.g. Fts proteins [32]) or peptidoglycan synthesis-associated proteins (e.g. PBP, Mur proteins [12]) we tested. Therefore, to determine if an epistatic relationship exists between Cls, MreB, and peptidoglycan synthesis, we performed a series of experiments leveraging the known effects of A22, an MreB inhibitor [41], and D-cycloserine (DCS), which blocks early steps in the synthesis of peptidoglycan precursors [42], on the localization of these markers. We previously used a similar strategy to demonstrate that MreB acts epistatically to penicillin binding proteins in *Chlamydia* [11].

**Fig 6:**
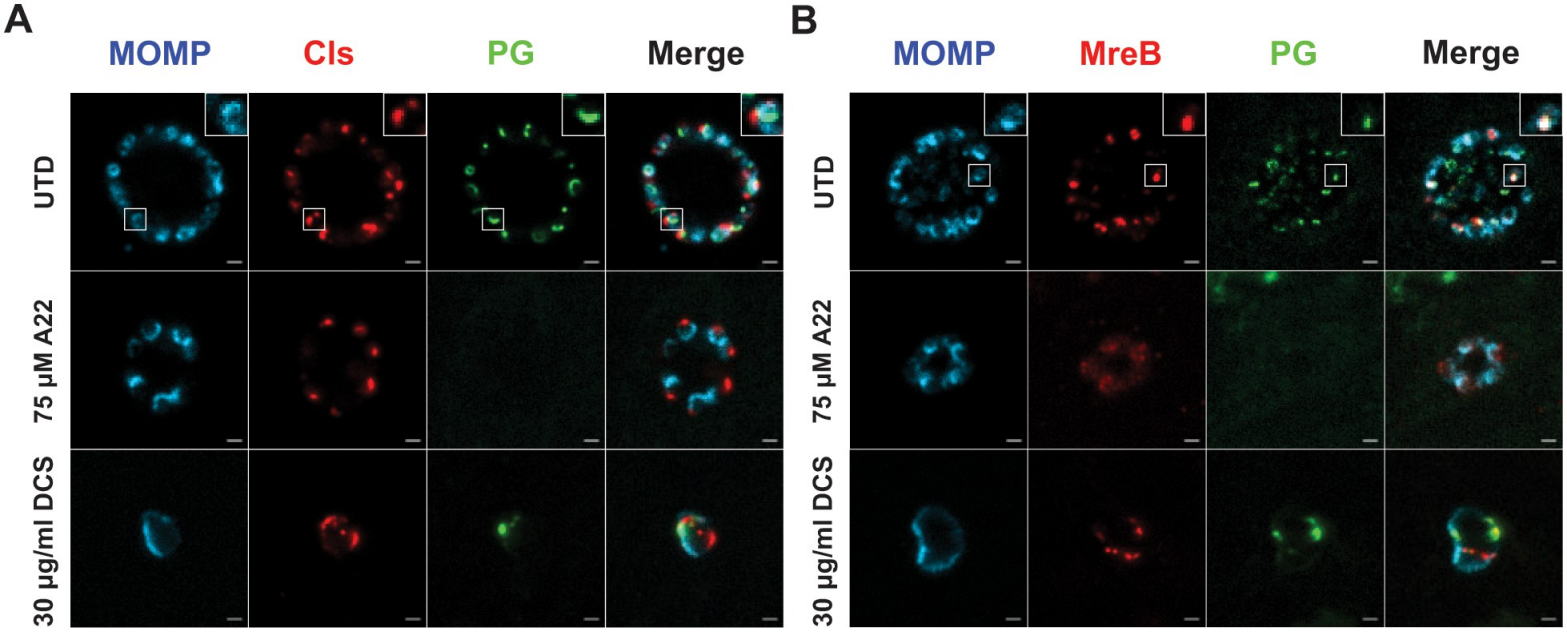
Effect of cell division inhibitors on localization of (A) Cls_6xH (labeled Cls) and (B) MreB_6xH (labeled MreB). Cells were infected with transformants carrying anhydrotetracycline (aTc) inducible vectors encoding the indicated constructs. Expression of Cls_6xH or MreB_6xH was induced at 16 hpi, and cells were fixed and processed for immunofluorescence at 20 hpi. To visualize peptidoglycan (PG), cells were incubated with EDA-DA during the infection and processed using click-iT reagents as described in the Materials and Methods. To assess effects of disrupting the central coordinator of chlamydial cell division, MreB, the antibiotic A22 was added at 18hpi whereas peptidoglycan synthesis was disrupted using D-cycloserine (DCS) added at 4hpi. Note the continued presence of Cls_6xH at a discrete site on the bacteria under all conditions tested. The insets within the upper row images are a zoomed-in view of the boxed regions in the larger image. Images were acquired on a Zeiss AxioImager.Z2 equipped with an Apotome2 using a 100X lens objective. Images are representative of at least three independent experiments. Scalebar = 2 μm.

To perform these experiments, cells were infected with chlamydial transformants carrying inducible expression constructs of Cls_6xH or MreB_6xH. Expression of the constructs was induced at 8hpi with aTc. DCS was added at 4hpi whereas A22 was added at 16hpi. Cells were labeled for peptidoglycan using the Click-iT modifiable EDA-DA prior to fixation at 18hpi and processing for immunofluorescence [43]. In induced samples without antibiotic treatment, the distinct polar localization of Cls_6xH was observed, and peptidoglycan rings and other peptidoglycan intermediates were detected (Fig 6A). However, these structures were generally not colocalized. MreB_6xH localization was consistent with prior studies, and the peptidoglycan labeling colocalized with MreB (Fig 6B). Interestingly, with A22 treatment, MreB_6xH localization was significantly more diffuse and peptidoglycan labeling was lost, consistent with earlier findings [13], but Cls_6xH remained localized at a discrete site on the membrane. DCS, while grossly altering bacterial cell shape and the distribution of peptidoglycan, did not affect the clustered membrane localization of Cls_6xH or MreB_6xH. These data indicate that clustered localization of Cls_6xH is not impacted by the inhibition of MreB or peptidoglycan synthesis.

One interpretation of our data could be that premade Cls_6xH is recruited to the membrane by MreB, but its continued presence there is not dependent on MreB. We tested this possibility by pre-treating cells with A22 to disrupt MreB localization prior to inducing expression of Cls_6xH. However, under these conditions, induced Cls_6xH was still recruited to a discrete location on the membrane (S9A and S9B Fig), indicating that its clustered distribution in cells occurs independently of MreB.

Since we could not stain infected cells with NAO to determine the effect of A22 treatment on the distribution of NAO-staining aPLs, we induced the expression of Cls_6xH by the addition of 10nM aTc to infected cells at 20hpi in the presence or absence of 75μM A22. Cells were lysed at 22hpi and the distribution of Cls_6xH and NAO-staining aPLs was assessed in the chlamydial cells in the lysate. The A22 treatment resulted in a round morphology for ~85% of the cells in the lysate. Therefore, we only assessed the localization profiles of Cls_6xH and NAO-staining aPLs in round cells (S9C Fig) prepared from drug treated and control samples. Quantification of the distribution profiles of Cls_6xH and NAO-staining aPLs revealed that both remained clustered following A22 treatment of the cells (S9C and S9D Fig). Similar analyses using uninduced conditions for the Cls_6xH transformant or wild-type *C*. *trachomatis* serovar L2 revealed that endogenous NAO-staining aPLs also remained clustered following A22 treatment (S9C and S9D Fig). In control experiments, we infected HeLa cells with an MreB_6xH transformant and induced the expression of MreB_6xH by the addition of 10nM aTc to infected cells at 20hpi in the presence or absence of 75μM A22. Cells were lysed at 22hpi and the distribution of MreB_6xH was assessed in chlamydial cells in the lysate. This analysis revealed that MreB_6xH was primarily in a polar cluster in round, pre-division intermediates, and shifted to a diffuse localization profile when cells were incubated in the presence of A22 (S9C and S9D Fig). These data indicate that A22 treatment disrupts the polar distribution of MreB_6xH in round cells and that the polar distribution of Cls_6xH and NAO-staining aPLs is not dependent upon MreB function.

### Assembly of MreB is Altered in diNN-treated *Chlamydia trachomatis*

**Fig 7:**
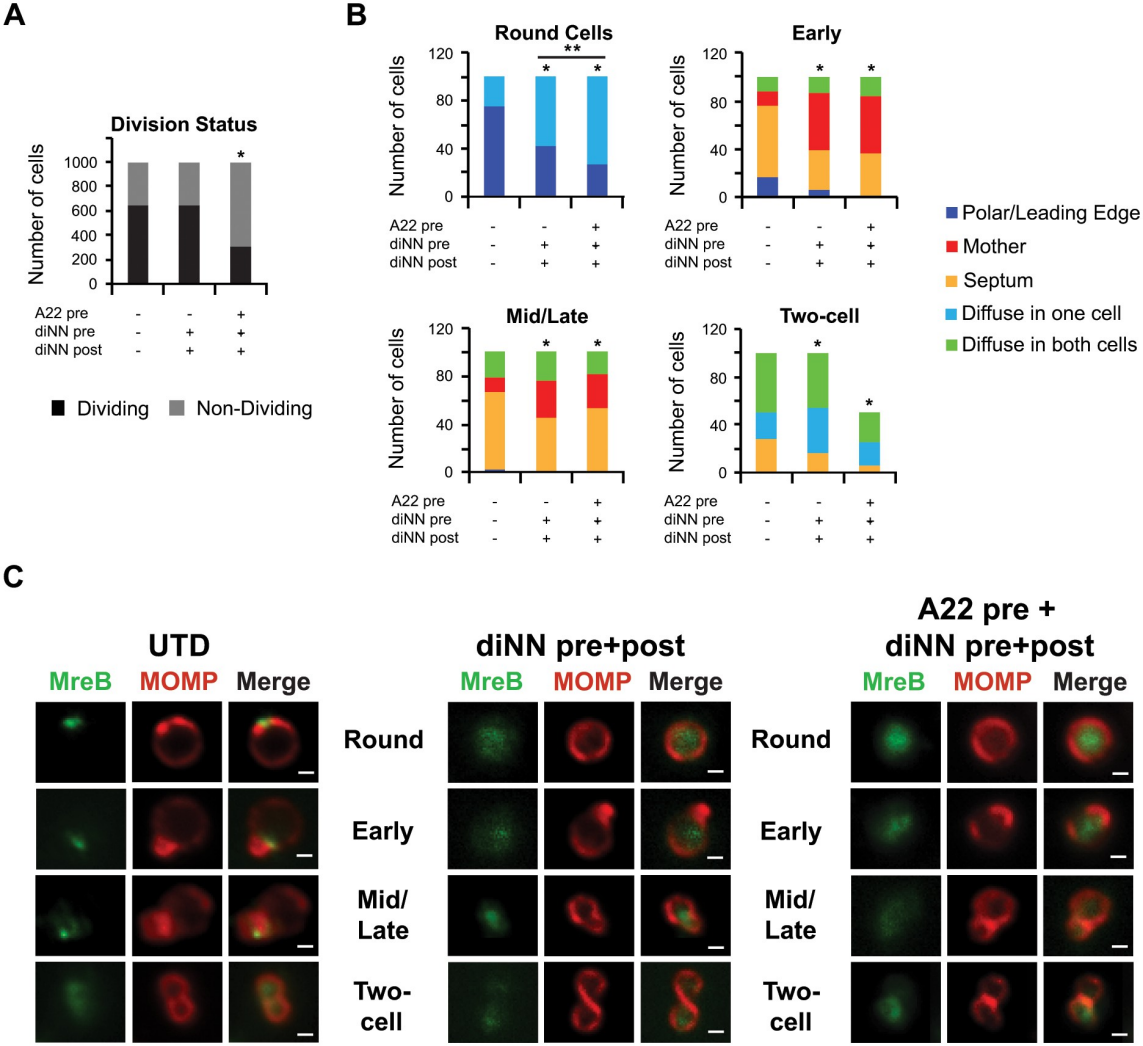
Effect of the CL-targeting antibiotic 3’,6-dinonylneamine (diNN) and the MreB-targeting antibiotic A22 on localization of MreB_6xH. HeLa cells were infected with the MreB_6xH transformant, and, at 22hpi, infected cells were lysed and chlamydial cells in the lysate were induced by incubating the cells in axenic media containing 10nM aTc. In some instances, cells were preincubated with 5μM diNN or 5μM diNN + 75 μM A22 for 30 minutes and then induced with aTc in the presence of 5μM diNN alone. Following induction, the localization of MreB_6xH at various stages of division was assessed by staining cells with MOMP and 6xHis antibodies. (A) The total number of dividing versus non-dividing cells from the indicated culture conditions was quantified from 1000 total bacteria. * = p<0.0001 compared to the untreated (UTD) control as measured by chi-squared test. (B) The localization of MreB_6xH was assessed in individual cells from each stage of division from untreated cultures or cultures treated as indicated in the figure. Localization profiles were categorized into leading edge of the budding daughter cell/polar, diffuse in mother cell, diffuse in one cell, diffuse in both cells, or septum. The differences in localization of MreB_6xH between treatment conditions at each stage of division were statistically analyzed using a chi-squared test to reveal that the changes resulting from drug treatments were statistically significant when compared to UTD (* = p<0.0001). Pretreatment of cells with diNN and A22 resulted in a statistically significant difference in MreB localization in pre-division intermediates (round cells) when compared to pretreatment with diNN alone (** = p<0.02). For (A) and (B), data were pooled from two independent experiments. (C) Representative images illustrating the distribution of MreB_6xH (labeled MreB) at different stages of division in untreated cells or in cells treated with drugs as indicated in the figure. Scalebar = 2 μm.

To directly investigate our hypothesis that membrane sub-domains enriched in CL are involved in directing MreB recruitment to specific sites in dividing *Chlamydia*, we investigated the effect of diNN on MreB_6xH localization in chlamydial cells incubated in axenic media. HeLa cells infected with a *Chlamydia* transformant carrying an aTc-inducible MreB_6xH plasmid were lysed at 22hpi, and the expression of MreB_6xH was induced in cells in the lysate by adding aTc to the cells incubated in axenic media for 1 hr. The effect of diNN on MreB_6xH assembly was determined by pretreating the chlamydial cells with 5μM diNN for 30 minutes then inducing MreB_6xH expression for 1hr in the continued presence of the drug. In addition, to eliminate any effects related to the preexisting endogenous pool of MreB recruiting exogenously expressed MreB_6xH, purified cells were pretreated with 5μM diNN plus 75μM A22 for 30 minutes prior to inducing MreB_6xH expression for 1 hour in the presence of diNN alone. For all treatment conditions, we quantified the number of dividing versus non-dividing cells in the population and observed that A22 pretreatment significantly reduced the overall number of dividing cells (Fig 7A), consistent with the central role of MreB in orchestrating the synthesis of PG in the septum to drive daughter cell outgrowth [13]. Conversely, diNN treatment did not significantly impact the ratio of non-dividing to dividing cells (Fig 7A).

We initially evaluated the localization of the ectopically expressed protein in cells induced in axenic media that were not treated with drug. Similar to what was observed when we induced MreB_6xH expression in cells during infection (S9C Fig), MreB_6xH was primarily in a polar cluster in pre-division “round” intermediates (Fig 7B and 7C). Although MreB_6xH exhibited a septal localization profile in the majority of early division intermediates, a subset of these intermediates retained MreB_6xH at the leading edge of the daughter cell, suggesting there is a slow transition of MreB_6xH from the leading edge of the daughter to the septum at this stage of division (Fig 7B and 7C). In mid/late budding intermediates, MreB_6xH was primarily septal, and it was mostly diffuse at the two-cell stage (Fig 7B and 7C). A very similar array of MreB_6xH localization profiles was observed when we induced the expression of MreB_6xH in infected cells at 21hpi, lysed the cells at 22hpi, and determined the distribution of the fusion protein in chlamydial cells in the lysate (S10 Fig). These results suggest that MreB_6xH, like Cls_6xH, transitions between different cellular compartments during the polarized budding process of *Chlamydia*.

To assess the effect of diNN on the localization profile of MreB_6xH, chlamydial cells in the lysate were pre-treated with 5μM diNN for 30 minutes in axenic media then induced with aTc for 1 hour in the continued presence of the drug. This drug treatment resulted in a diffuse pattern of localization of MreB_6xH in the majority of round, early, and mid/late division intermediates (Fig 7B and 7C). Quantification of these analyses revealed a statistically significant effect of diNN on the distribution of MreB_6xH at each stage of division compared to the untreated control (Fig 7B). These results indicate that the localized assembly of MreB_6xH in dividing chlamydiae is dependent upon the maintenance of CL-rich membrane microdomains.

It was possible that a preexisting pool of endogenous polymeric MreB serves as a nucleation site for the assembly of newly synthesized MreB_6xH in a subset of the dividing cells treated with diNN. Therefore, in addition to diNN, we also pretreated cells with A22, to depolymerize this preexisting pool of endogenous MreB. The A22 was then removed and MreB_6xH was induced for 1 hour in the continued presence of diNN. Although there was a reduction in the number of cells in the population undergoing division when they were treated in this way (Fig 7A), the results observed in those cells undergoing division were similar to the treatment with diNN alone (Fig 7A–7C). However, pretreatment with A22 resulted in a further significant reduction in the number of round (pre-division) intermediates with polar MreB_6xH, suggesting that, at least at this stage of division, a preexisting pool of polymeric MreB may serve as a nucleation site for the assembly of newly synthesized MreB_6xH in the presence of diNN. Taken together, our results indicate a critical role for CL-rich membrane domains in directing the localized assembly of MreB_6xH in round, early, and mid/late division intermediates. Furthermore, the formation of these CL-rich membrane microdomains depends upon the polarized synthesis of CL by Cls.

## Discussion

In model organisms like *E*. *coli* or *B*. *subtilis* that use an FtsZ-dependent binary fission mechanism to divide, there are conserved systems to ensure proper placement of the FtsZ ring in the middle of the cell. These include, for example, the MinCDE and nucleoid occlusion (Noc) systems [44–47]. *Chlamydia* has no annotated or otherwise obvious orthologs to these proteins (ParA, a chromosome partitioning ATPase that *Chlamydia* encodes, shares homology with MinD) [10]. *Chlamydia* is unique amongst characterized bacteria in using an MreB-dependent polarized cell division process [11,16]. Although much recent work has identified the predicted components of the chlamydial divisome [11,12,14,32], it is unclear how a division site is selected given that *Chlamydia* are coccoid bacteria with apparent uniformity in their negative membrane curvature. An additional issue for *Chlamydia* is that MreB is typically excluded from areas of negative membrane curvature, thus how it is recruited to the inner leaflet of the inner membrane at a distinct site was unclear prior to this study.

**Fig 8:**
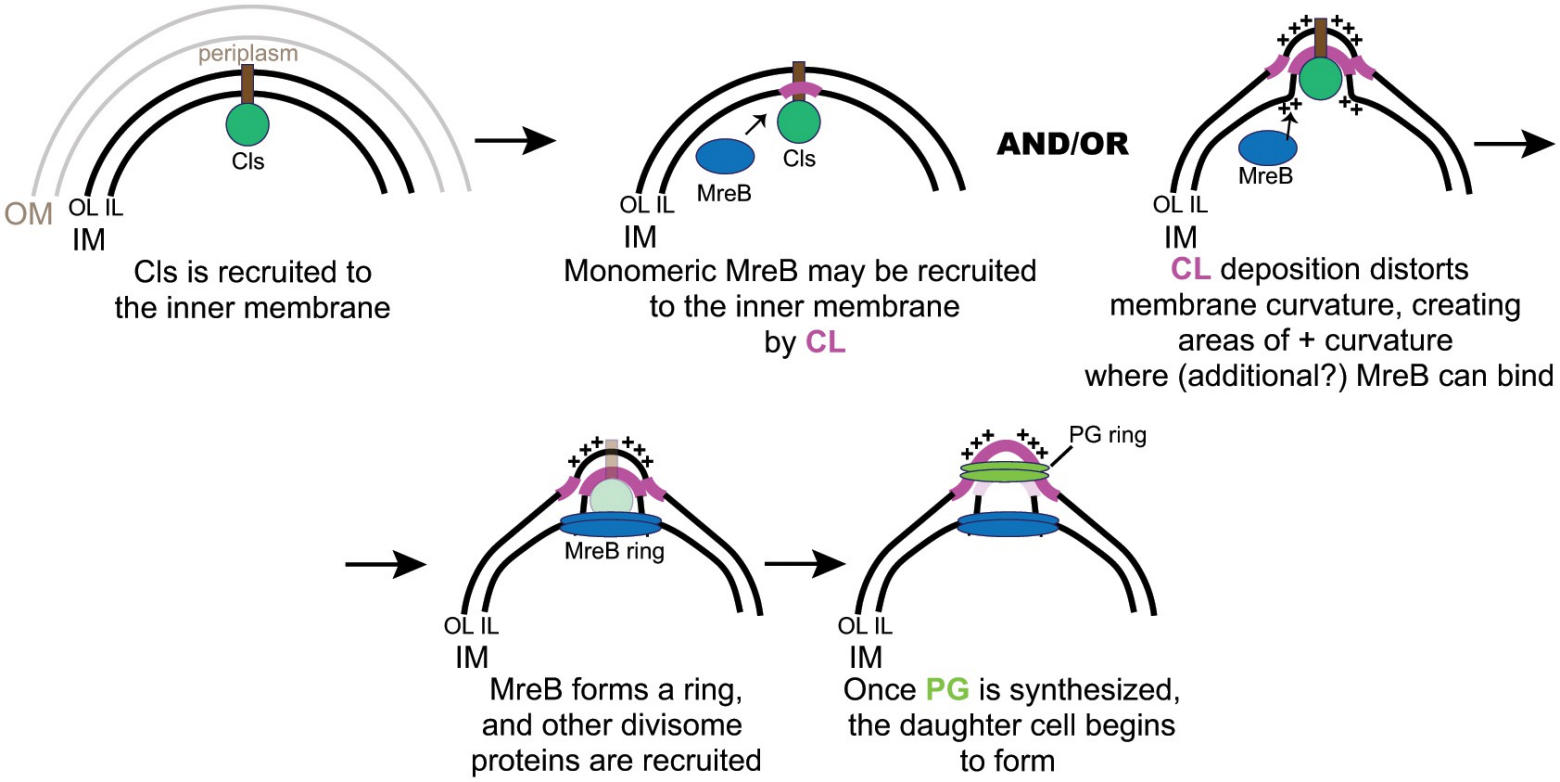
Model for the function of cardiolipin (CL) and Cls in chlamydial cell division. How Cls is recruited to the membrane at a distinct site is not understood but requires the transmembrane domain. An initial deposition of CL may either directly recruit MreB or induce membrane curvature that recruits MreB, and these are not mutually exclusive. Once MreB forms a ring, it recruits subsequent cell division machinery to allow peptidoglycan (PG) synthesis in the periplasm. OM = outer membrane; IM = inner membrane; OL = outer leaflet of IM; IL = inner leaflet of IM; ++ = areas of positive membrane curvature in each leaflet of the IM.

Our data indicate that CL-rich membrane domains are necessary for the localized assembly of MreB_6xH in pre-(round), early, and mid/late division intermediates. We hypothesize that this polar population of CL either directly recruits MreB or drives localized membrane deformation to enable MreB recruitment to the site where the daughter cell will bud (modeled in Fig 8). CL has a small head group with 4 acyl chains that allows it to be packed into areas of high curvature [24]. Although seemingly counterintuitive, localized deposition of CL in the inner leaflet would induce membrane bending that necessarily creates areas of *positive* curvature in the paired outer leaflet of the membrane and in the areas directly flanking the inner leaflet where CL was deposited. Such areas would favor MreB recruitment and subsequent peptidoglycan synthesis to initiate the division process. Continued outgrowth of the budding daughter cell would, however, necessitate CL in the outer leaflet at the “junction” where the daughter cell arises from the mother cell since this area will also have curvature constraints.

To test how a polarized distribution of CL could arise in *Chlamydia*, we assessed various parameters associated with localization of the chlamydial CL synthase. These studies were dependent upon the overexpression of CL synthase in *Chlamydia*, and we have shown that the distribution of CL synthase and NAO-staining aPLs in cells where Cls_6xH expression was induced mimics the distribution of endogenous aPLs in *Chlamydia*. While this result does not exclude the possibility that aspects of chlamydial cell division were influenced by our overexpression model, Cls_6xH overexpression does not appear to impact the distribution of aPLs in dividing *Chlamydia* and has no effect on DNA replication rates during infection. At the onset of division, Cls_6xH exhibited a polarized pattern of localization at the side of the cell where the daughter cell forms. Cls_6xH remained associated with the leading edge of the daughter cell at later stages of the division process in support of our model. The restricted localization of Cls_6xH overlapped the distribution of NAO-labeled aPLs, and Cls_6xH and NAO-labeled aPLs were redistributed to the mother cell by diNN, a drug that disrupts CL-rich membrane microdomains. Importantly, the localization of a neutral phospholipid (phosphatidylethanolamine; PE) synthase, PsdD_6xH, was restricted to the mother cell membrane distal to the division site, and its localization was unaffected by diNN. These data suggest that general PL membrane synthesis may be restricted to the mother cell during the division process, which is consistent with the function of PE as a major cell membrane constituent in *Chlamydia* [25,26]. Conversely, the deposition of CL is restricted to a polarized site in *Chlamydia* associated with the dividing (daughter) side of the cell. It remains unclear at this time if the recruitment of Cls to a polarized site is regulated or if its insertion into the membrane creates spontaneous symmetry breaking that, in turn, locks in the polarization of the organism. Given the polarity observed in the EB form [16], it is likely the former, but our data cannot currently discriminate between these possibilities.

Our model predicts that Cls acts epistatically to MreB and other divisome machinery in the division process. Our data support such a model. Restricted localization of Cls_6xH either before or after treating infected cells with the MreB or PG-disrupting drugs, A22 or D-cycloserine (D-CS), respectively, was not altered. Although MreB_6xH membrane localization was disrupted by A22, as previously noted [13], with a concomitant loss of PG signal, A22 did not affect the clustered localization of NAO-staining aPLs in chlamydial cells. D-CS, as expected [43], did not impact MreB_6xH but did disrupt PG ring formation, resulting in aberrantly enlarged organisms. Overall, these data suggest that Cls is epistatic to MreB during cell division. Moreover, our inability to detect a physical interaction between Cls and divisome components by two hybrid assays suggests that it is the activity of Cls (i.e. generating CL) and not its ability to recruit specific protein factors that is important for its function in any cell division context. Importantly, our results with diNN-treated *Chlamydia* support our proposed model and suggest that the establishment of CL-rich membrane microdomains is critical for the localized assembly of MreB during chlamydial division.

It is not clear on which side of the inner membrane CL exerts its function as PLs can be flipped across membranes [48]. Importantly, our model posits a function for CL on *both* leaflets depending on the stage of daughter cell outgrowth. Determining lipid localization, particularly to a single leaflet is difficult, and the small size of chlamydial RBs preclude using light-based microscopy techniques to address this question. To investigate the distribution of CL in the chlamydial membrane, we expressed a characterized mitochondrial CL binding protein, C11orf83 [29], within different compartments of *Chlamydia*. Of note, C11orf83 also binds phosphatidic acid (PA) [29], which is present at low levels as a short-lived PL precursor in bacteria that is incorporated into CDP-diacylglycerol in the PL synthesis pathway [37,38]. The full-length C11orf83 exhibited a diffuse localization profile in *Chlamydia* and had no obvious impact on chlamydial growth or morphology. In contrast, by tethering the full-length C11orf83 to the inner leaflet of the inner membrane using the Cls TM domain, there was a dramatic and negative impact on *Chlamydia*. However, given the possibility that C11orf83 binds PA in the inner leaflet, we cannot with absolute certainty assign the negative impact on *Chlamydia* to disruption of CL. Strikingly, when C11orf83 was tethered to the outer leaflet of the inner membrane within the periplasm, this severely disrupted chlamydial growth and morphology as well. We are unaware of any reports demonstrating free PA in the outer leaflet of the inner membrane or the periplasm more generally in bacteria, thus it is likely that the negative impacts of C11orf83 in the periplasm are due to its binding of CL. This was not a general effect associated with overexpressing a protein in the periplasm since tethering mCherry to the outer leaflet of the inner membrane had much less of an effect on chlamydial growth. Collectively, these data suggest a critical function for CL in both leaflets of the inner membrane and support our model.

Other published data related to divisome components and PG are consistent with our proposed model that an initial deposition of MreB and PG, driven by localized CL synthesis in the membrane, occurs before MreB or PG rings are observed. For example, we show here that MreB_6xH forms a polarized cluster at the MOMP-enriched side of the cell prior to division and a ring at the base of the daughter cell as it grows. In addition, we demonstrated specific functions for the PG transpeptidases PBP2 and PBP3 in daughter cell growth, showing that inhibition of PBP2 prevents the initiation of daughter cell formation with only a patch of polarized PG detectable underneath the MOMP label in treated organisms [17]. MreB is epistatic to PBP2 and PBP3 [11].

How polarity is established in *Chlamydia* is an interesting question. In other organisms, specific protein factors like bactofilins have been associated with polar localization. Interestingly, *Chlamydia* has a bactofilin ortholog, but, as we recently characterized, it is not associated with division but rather with cell size [49]. Therefore, further work is required to understand how Cls is recruited to a discrete site and whether specific protein factors or other context-dependent cues drive its localization. Other questions under investigation in our labs include how membrane synthesis changes as the daughter cell grows and how these changes are resolved at the completion of the division process. Given the unique biology of *Chlamydia*, it is possible that they have once again co-opted conserved systems for their specific needs.

## Materials and methods

### Organisms and Cell Culture

HeLa (ATCC, Manassas, VA), HEp2, and McCoy (kind gift of Dr. Harlan Caldwell) cells were cultured in Dulbecco’s Modified Eagle Medium (DMEM; Invitrogen, Waltham, MA) containing 10% fetal bovine serum (FBS; Hyclone, Logan, UT) and 10 μg/mL gentamicin (Gibco, Waltham, MA) at 37°C with 5% CO_2_. Wild-type *C*. *trachomatis* serovar L2/434/Bu was cultured in HeLa cells, and EBs were purified from cell lysates through a renografin density gradient as described elsewhere [50]. *Chlamydia trachomatis* serovar L2 lacking the endogenous plasmid (-pL2) (kind gift of Dr. Ian Clarke) was infected and propagated in McCoy cells for use in transformations. HeLa cells were infected with chlamydial transformants in DMEM containing 10% FBS, 10 μg/mL gentamicin, 1 U/mL penicillin G, and 1 μg/mL cycloheximide. All cell cultures and chlamydial stocks were routinely tested for Mycoplasma contamination using the Mycoplasma PCR detection kit (Sigma, St. Louis, MO). All chemicals and antibiotics were obtained from Sigma unless otherwise noted.

### Cloning

The plasmids and primers used in this study are described in S1 Table. The wild-type or mutant chlamydial *cls*, *psdD*, and *mreB* genes were amplified by PCR with Phusion DNA polymerase (NEB, Ipswich, MA) using 10 ng *C*. *trachomatis* L2 genomic DNA as a template. Some gene segments were directly synthesized as a gBlock fragment (Integrated DNA Technologies, Coralville, IA). The PCR products were purified using a PCR purification kit (Qiagen, Hilden, Germany). The HiFi Assembly reaction master mix (NEB) was used according to the manufacturer’s instructions in conjunction with plasmids pBOMB4-Tet (kind gift of Dr. Ted Hackstadt [51]) cut with EagI and KpnI or the BACTH vector pSTM25 digested with KpnI and BamHI [39]. All plasmids were dephosphorylated with alkaline phosphatase (FastAP; ThermoFisher) prior to use in the HiFi reaction. The products of the HiFi reaction were transformed into NEB-10β (chlamydial transformation) or NEB-5αI^q^ (BACTH) competent cells (NEB), plated on appropriate antibiotics, and plasmids were subsequently isolated from individual colonies grown overnight in LB broth by using a mini-prep kit (Qiagen). All plasmids were verified for correct size by digest, and inserts were sequenced.

### Bioinformatics analysis

Sequences for *Chlamydia trachomatis* serovar L2/434, different chlamydial species, and *Bacillus subtilis* were obtained from the NCBI database (Available from: https://www.ncbi.nlm.nih.gov/) and for *E*. *coli* MG1655 from Ecocyc database (Available from: https://ecocyc.org/) [52]. Protein sequence alignment was performed using Clustal Omega website (Available from: https://www.ebi.ac.uk/Tools/msa/clustalo/) [53] and the ESPript3 program (Available from: http://espript.ibcp.fr) [54]. TOPCONS [55] and TMHMM [56] were used for transmembrane domain prediction.

### Nucleic acid extraction and RT-qPCR

Total RNA and DNA were extracted from *C*. *trachomatis* L2/434Bu-infected HEp2 cells plated in 6-well dishes as described previously [57]. Briefly, for RNA, cells were rinsed one time with PBS, then lysed with 1mL Trizol (Invitrogen) per well. Total RNA was extracted from the aqueous layer after mixing with 200μL per sample of chloroform following the manufacturer’s guidelines. Total RNA was precipitated with isopropanol. Purified RNA was rigorously DNased using DNAfree (Ambion) according to the manufacturer’s guidelines prior to synthesis of cDNA using SuperScript III (Invitrogen) following the manufacturer’s guidelines. For DNA, cells were rinsed one time with PBS, trypsinized, and pelleted before resuspending each pellet in 500μL PBS. Each sample was split in half (i.e. 250μL), and genomic DNA was isolated from each duplicate sample using the DNeasy extraction kit (Qiagen) according to the manufacturer’s guidelines.

Quantitative PCR to detect genomic DNA (gDNA) levels of *C*. *trachomatis* using an *euo* primer set and RT-qPCR to detect *cls* transcript levels were performed as described previously using SYBR Green (see S1 Table for primer sequences) [57]. For gDNA levels, 150ng of each sample was used in 25μL reactions using standard amplification cycles on a Quantstudio3 thermal cycler (Applied Biosystems) followed by a melting curve analysis. For cDNA levels, equal volumes of each cDNA reaction were used in 25μL reactions under standard amplification cycle conditions with melting curve analysis. Transcript levels were normalized to genomes and expressed as ng cDNA/gDNA.

### Transformation of *Chlamydia trachomatis*

McCoy cells were plated in a six-well plate at a density of 1 x 10^6^ cells per well the day before beginning the transformation procedure. *C*. *trachomatis* serovar L2 lacking its endogenous plasmid (-pL2) was incubated with 2 μg plasmid in Tris-CaCl_2_ buffer (10 mM Tris-Cl pH 7.5, 50 mM CaCl_2_) for 30 min at room temperature [6,8]. During this step, the McCoy cells were washed with 2 mL 1X Hank’s Balanced Salt Solution (HBSS) media containing Ca^2+^ and Mg^2+^ (Gibco). After that, McCoy cells were infected with the transformants in 2 mL HBSS per well. The plate was centrifuged at 400 x g for 15 min at room temperature and incubated at 37°C for 15 min. The inoculum was aspirated, and DMEM containing 10% FBS and 10 μg/mL gentamicin was added per well. At 8 h post infection (hpi), the media was changed to media containing 1 μg/mL cycloheximide and 1 or 2 U/mL penicillin G, and the plate was incubated at 37°C until 48 hpi. At 48 hpi, the transformants were harvested and used to infect a new McCoy cell monolayer. These harvest and infection steps were repeated every 48 hpi until mature inclusions were observed. DNA was isolated from transformants using a Genomic DNA isolation kit (DNeasy; Qiagen) and used to transform NEB-10β competent cells to reverify the plasmid as described above for size and insert sequence fidelity.

### Indirect Immunofluorescence (IFA) Microscopy

HeLa cells were seeded in 24-well plates on coverslips at a density of 1.5 x 10^5^ cells per well the day before infection. Chlamydial strains expressing wild-type Cls or MreB with a six-histidine tag at the C-terminus were used to infect HeLa cells in DMEM media containing 1 U/ml penicillin G and 1 μg/mL cycloheximide. Anhydrotetracycline (aTc) was added at the indicated concentration at the indicated time. The coverslips of infected cells were washed with 1X DPBS and fixed with fixing solution (3.2% formaldehyde and 0.022% glutaraldehyde in 1X DPBS [16]) for 2 min at various times during infection. To observe the effect of A22 and D-cycloserine (DCS), we added 75 μM A22 or 30 μg/mL DCS at 18 hpi or 4 hpi, respectively. The samples were then washed three times with 1X DPBS and permeabilized with ice-cold 90% methanol for 1 min. Afterwards, the fixed cells were labeled with primary antibodies including goat anti-major outer-membrane protein (MOMP; Meridian, Memphis, TN), rabbit anti-chlamydial MreB antibody (custom anti-peptide antibody directed against the C-terminus of *C*. *trachomatis* serovar L2 MreB; ThermoFisher), or rabbit anti-six histidine tag (Genscript, Piscataway, NJ, and Abcam, Cambridge, MA, respectively) as indicated. Donkey anti-goat (647 or 488) or donkey anti-rabbit (594) secondary antibodies (Invitrogen) were used to visualize the primary antibodies. Coverslips were observed by using either a Nikon Ti2 spinning disc confocal microscope using a 60X lens objective or a Zeiss AxioImager.Z2 equipped with an Apotome2 using a 100X lens objective.

### Peptidoglycan (PG) staining

PG was labelled with D-amino acid dipeptide probes and click chemistry as previously described [43]. Briefly, HeLa cells were infected with *C*. *trachomatis* containing an anhydrotetracycline (aTc)-inducible vector encoding chlamydial Cls_6xH or MreB_6xH. 1 mM EDA-DA, which is the D-amino acid dipeptide probe, was added at 0 hpi. The constructs were induced with 10 nM anhydrotetracycline (aTc) at 16 hpi. At 20 hpi, cells were washed 3 times with 1X DPBS and fixed with 100% methanol for 5 min. After washing the samples three times with 1X DPBS, the cells were permeabilized with 0.5% Triton X-100 for 5 min. The samples were blocked with 3% Bovine Serum Albumin (BSA) for 1 hour. The PG was labeled by using the Click-iT cell reaction buffer kit according to the manufacturer’s instructions (Invitrogen, Waltham, MA).

### Inclusion forming unit assays

HeLa cells were infected with C. *trachomatis* serovar L2 transformed with a plasmid encoding an aTc-inducible gene as indicated. At 8 or 16hpi, aTc was added to the culture media at the indicated concentration. At 24 hpi the HeLa cells were dislodged from the culture dishes by scraping and collected into microcentrifuge tubes. Suspensions were centrifuged at 4°C for 30 min, the supernatant was removed by aspiration, and the pellet was resuspended in 1mL 2 sucrose-phosphate (2SP) solution [6] and frozen at -80°C. At time of secondary infection, the chlamydiae were thawed on ice and vortexed. Cell debris was pelleted by centrifugation for 5 min at 1k x g, 4°C. The *C*. *trachomatis* elementary bodies (EBs) in the resulting suspension were serially diluted and used to infect a monolayer of HeLa cells in a 24-well plate. The secondary infection was allowed to grow at 37°C with 5% CO_2_ for 24 h before they were fixed, labeled for immunofluorescence microscopy with goat anti-MOMP antibody and a secondary donkey anti-goat antibody labeled with Alexa Fluor 594, and counted. Titers were enumerated by calculating the total number of inclusions per field based on counts from 20 fields of view. Three independent replicates were performed, and the totals for each experiment were averaged. Results were normalized as a percentage of the uninduced samples for each time point. Student’s two-tailed parametric, unpaired t tests were used to compare the induced samples to the uninduced samples in GraphPad Prism 9 using the averages of each biological replicate.

### Localization of Cls_6xH and anionic phospholipids in *C*. *trachomatis*

HeLa cells were infected with C. *trachomatis* serovar L2 or C. *trachomatis* serovar L2 transformed with a plasmid encoding an aTc-inducible Cls_6xH. At 20hpi, 10nM aTc was added to the culture media, and the HeLa cells were then harvested from the culture dishes by scraping at 22hpi. The HeLa cells were pelleted, and *Chlamydia* were released from infected cells as described previously [6]. Briefly, infected HeLa cells were resuspended in 1mL of 0.1x PBS and vortexed in tubes containing 0.1mM glass beads. The lysate was centrifuged at 1000rpm for 3 minutes and 20μL of the supernatant was transferred to a glass slide and mixed with an equal volume of 2x fixative (6.4% formaldehyde, 0.044% glutaraldehyde in PBS) and incubated for 10 min at room temperature. The cells were washed 3x in PBS and permeabilized by incubation with PBS containing 0.1% TX-100 for 1 minute. Following washing in PBS, the cells were incubated with goat polyclonal antibodies directed against MOMP and rabbit polyclonal antibodies directed against 6xHis tag (Abcam) for 1 hour. The cells were washed and incubated with donkey anti-goat IgG conjugated to Alexa 594 and donkey anti-rabbit IgG conjugated to Alexa 488. Cells were again washed and imaged using Zeiss AxioImager2 microscope equipped with a 100x oil immersion PlanApochromat lens. Images were deconvolved using the deconvolution algorithm in the Zeiss Axiovision 4.7 software. Alternatively, aTc-induced cells labeled with MOMP antibodies were incubated with 250nM NAO for 1 hour (Invitrogen) to visualize the distribution of anionic phospholipids following Cls_6xH induction. In similar analyses, HeLa cells were infected with wild-type C. *trachomatis* serovar L2 or the Cls_6xH transformant that was not induced. At 22hpi, *Chlamydia* were released from the infected cells and fixed and permeabilized as described above. The distribution of endogenous anionic phospholipids relative to MOMP was assessed by incubating the fixed cells with 250nM NAO for 2 hours. In some instances, the distribution of Cls_6xH and NAO staining were visualized in the same cell that was also stained with HOECHST 33342 to visualize DNA.

Z-stacks were collected during image acquisition that extended above and below the dividing cell. The largest diameter of the nascent daughter cell and the progenitor mother cell was determined using the measuring tool in the Zeiss AxioVision 4.7 software. This value was used to estimate the volume of the daughter and mother cell according to the formula, v = 4/3πr^3^. For all experiments we defined several intermediates in the polarized division process. Pre-division intermediates (round cells) had no obvious outgrowth of the budding daughter cell, in early division intermediates the daughter cell volume was <15% of the mother cell volume, in mid/late division intermediates the daughter cell volume was between 15–80% of the mother cell volume, and in the two-cell stage the daughter cell volume was >80% of the mother cell volume.

### Effect of the amphiphilic aminoglycoside, 3’,6-dinonylneamine (diNN), on the localization of Cls_6xH and anionic phospholipids in *C*. *trachomatis* incubated in axenic media

HeLa cells were infected with C. *trachomatis* serovar L2 transformed with a plasmid encoding an aTc-inducible Cls_6xH. At 22hpi, the HeLa cells were harvested as described above, and cells were lysed by vortexing in 1mL of 0.1x PBS in tubes containing 0.1mM glass beads. The lysate was centrifuged at 1000rpm for 3 minutes and 20 μL of the supernatant was transferred to a glass slide where it was mixed with 100μL of axenic media composed of Opti-MEM (Gibco), a minimal essential media containing insulin, transferrin, hypoxanthine, and thymidine that was supplemented with 1mM L-Glutamine, 1mM glucose-6-phosphate (Moltox), and 10nM aTc. The slide was transferred to a humidified chamber and incubated at 37 °C for 1.5 hours in a CO_2_ incubator. The cells were then fixed by mixing the cells with an equal volume of 2x fixative and incubating for 10 min at room temperature. Fixed cells were permeabilized by incubating in PBS containing 0.1% TX-100 for 1 min, and the localization of Cls_6xH and anionic phospholipids in aTc-induced cells was determined as described above. To determine the effect of the amphiphilic aminoglycoside, diNN, on the localization of Cls_6xH and anionic phospholipids, 5μM diNN was present in the axenic media throughout the aTc induction.

### Effect of 3’,6-dinonylneamine (diNN) and A22 on the localization of MreB_6xH in *C*. *trachomatis* grown in axenic media

HeLa cells were infected with C. *trachomatis* serovar L2 transformed with a plasmid encoding an aTc-inducible MreB_6xH. At 22hpi, *Chlamydia* were lysed and chlamydial cells in the lysate were incubated in axenic media containing 10nM aTc for 1 hour at 37°C in a CO_2_ incubator. The cells were then fixed and the distribution of MreB_6xH in dividing cells was assessed by staining with MOMP and 6xHis antibodies. The effect of 5μM diNN and 75μM A22 on MreB_6xH localization was assessed by pretreating cells with diNN alone or diNN + A22 for 30 minutes prior to a one-hour induction with aTc in the presence of diNN alone.

### Quantification of Cls_6xH, MreB_6xH, and NAO staining profiles in *C*. *trachomatis*

*C*. *trachomatis* in which Cls_6xH or MreB-6xH was induced were stained with MOMP antibodies and the 6xHis-tag antibody or NAO. In some instances, the distribution of endogenous NAO-staining anionic phospholipids was determined in cells where the expression of the fusion protein was not induced or in non-transformed, wild-type *C*. *trachomatis* serovar L2. For each condition, the Cls_6xH, MreB_6xH, and NAO staining profiles were imaged in at least 50 cells for each stage of division. For some of the drug treatments fewer than 50 cells were counted because certain division intermediates were present at very low levels following drug treatment. Localization profiles for the Cls_6xH, MreB_6xH, and NAO were categorized and the effect of the drugs on the localization profiles determined and statistically analyzed using a chi-squared test.

### Western blotting for C11orf83 expression levels

McCoy cells were infected with the transformants carrying aTc-inducible plasmids encoding C11orf83_6xH, Cls_TM-c11orf83_6xH, or OppB_TM-c11orf83_6xH. At 16 hpi, the constructs were induced with 2 or 10 nM aTc, and the infected cells were harvested at 24 hpi. The cells were resuspended with lysis buffer (50 mM Tris-Cl pH 7.4, 150 mM NaCl, 0.1% SDS, 0.5% sodium deoxycholate, 1X Halt protease inhibitor cocktail, 5 mM EDTA, 150 μM clasto-lactacystin β-lactone, and universal nuclease) and rotated for 20 min at 4°C at 10 rpm. The cell-debris and insoluble fraction were removed by centrifugation at 14,000 rpm for 30 min. The supernatant was removed to a new microcentrifuge tube, and the concentration was measured by EZQ protein assay kit (Thermo) following the manufacturer’s instructions. 100 μg crude extract was separated by 15% SDS-PAGE and electrophoretically transferred to a PVDF membrane (Thermo). To detect six histidine tagged constructs, the membrane was blotted with mouse anti-6xH (Genscript, Piscataway, NJ) and IRDye 800CW Donkey anti-Mouse IgG Secondary Antibody (LICOR, Lincoln, NE). To detect MOMP, we used goat anti-MOMP (Meridian, Cincinnati, OH) and IRDye 680RD Donkey anti-Goat IgG Secondary Antibody (LICOR). The membrane was imaged using an Azure c600 imager system (Azure Biosystems, Dublin, CA).

## Supporting information

S1 Table List of primers and plasmids used in the current study. (PDF)

S1 Fig (A) Alignment of *ct284/cls* orthologs from multiple pathogenic chlamydial species. The numbering is for the *C*. *trachomatis* protein. The predicted transmembrane domain is boxed in green with the catalytic sites boxed in black. White lettering on red background indicates conservation of the residues across all species whereas red lettering on white background indicates similarity at that position. (B) Transcriptional analysis of *cls* during the *C*. *trachomatis* L2 developmental cycle. Cells were infected with wild-type *C*. *trachomatis* L2/434Bu, and total RNA and DNA were collected at the indicated time points. Transcript levels were normalized to genomic DNA levels and are expressed as ng cDNA/gDNA. Shown are data from one of two experiments.
(PDF)

S2 Fig The localization of MreB at the round (pre-division) and mid/late stages of division. HeLa cells were infected with the transformant carrying an aTc-inducible plasmid encoding MreB_6xH. At 4 hpi, the construct was induced with 10 nM aTc, and the cells were fixed at 10.5 hpi. Images were acquired on a Nikon Ti2 spinning disc confocal microscope using a 60X lens objective. Images are representative of at least three independent experiments. Scalebar = 2 μm.
(PDF)

S3 Fig Effect of Clx_6xH overexpression on chlamydial growth. (A) Localization of wild-type Cls_6xH (labeled Cls) in *C*. *trachomatis* L2. Cells were infected, induced or not for expression at 4hpi with 5nM anhydrotetracycline (aTc), and samples were collected and processed at 24hpi. Bacteria were labeled with an antibody targeting MOMP (major outer membrane protein—green), and the construct was labeled with an antibody against the 6xH tag (red). Nuclear DNA was labeled with DAPI and is visualized within the merged image only in the blue channel. The boxed region within each induced image is shown below as an enlarged inset. Images were acquired on a Zeiss AxioImager.Z2 equipped with an Apotome2 using a 100X lens objective. Images are representative of at least three independent experiments. Scalebar of full inclusion images = 2 μm. Scalebar of inset = 0.5 μm. (B) Cells were infected with Cls_6xH transformant and processed as described in the Materials and Methods to quantify IFU production during the primary infection. The uninduced values were arbitrarily set to 100%, and the effect of overexpression at 5nM aTc when added at 8hpi is expressed as a percentage of the wild type. Data are the average of at least three biological replicates assayed in triplicate. *** = p = 0.0007. (C) Quantification of genomic DNA over a time course of infection for the Cls_6xH transformant in uninduced and induced (5nM aTc) conditions. Cell lysates were collected at the indicated time points, and genomic DNA was extracted and quantified by qPCR as described in Materials and Methods. No statistical differences were observed between the uninduced and induced conditions for any of the time points assessed. Data are the average of three independent experiments assayed in triplicate. (D) Cells were infected with Cls_6xH transformant, expression was induced or not at 16hpi with 10nM aTc, and IFUs were quantified at 24hpi. The uninduced values were arbitrarily set to 100%, and the effect of overexpression at 10nM aTc when added at 16hpi is expressed as a percentage of the wild type. Data are the average of at least three independent biological replicates assayed in triplicate. **** = p<0.0001. (E) The effect of Cls_6xH induction on the distribution of chlamydial division intermediates. HeLa cells infected with the Cls_6xH transformant were induced for 2hrs with 10nM aTc at 20hpi or were uninduced. The infected cells were lysed at 22hpi, fixed and stained with MOMP antibodies. The division intermediates (round, early, mid/late, and two-cell stages) in induced or uninduced cells were quantified. 500 total cells were counted from two independent experiments for each condition, and chi-squared analysis of the data revealed that the induction of Cls_6xH did not have a statistically significant effect on the profile of division intermediates in the population.
(PDF)

S4 Fig Localization of endogenous aPLs in dividing *Chlamydia*. HeLa cells were infected with the Cls_6xH transformant that was not induced (A and B) or with wild-type C. *trachomatis* serovar L2 (C and D). At 22hpi, *Chlamydia* were released from infected cells and the distribution of (A and C) MOMP and endogenous aPLs (stained with 250nM NAO for 2 hours) within chlamydial cells in the lysate was assessed. Representative images illustrating the localization profiles for each marker in Round cells (pre-division intermediates), and in the Early, Mid/Late, and Two-cell stages of the polarized division process are shown. The distribution of (B and D) MOMP and endogenous aPLs was evaluated in 50 individual cells from the indicated stages of division. Localization profiles for endogenous NAO-staining phospholipids were categorized into leading edge of the budding daughter cell/polar, diffuse in mother cell, diffuse in one cell, diffuse in both cells. Chi-squared analysis revealed that the localization profiles of endogenous aPLs in the uninduced Cls_6xH transformant (B) were not statistically different than the localization profiles of aPLs in cells where Cls_6xH expression was induced by the addition of 10nM aTc to the media of infected cells (Fig 2C). Chi-squared analysis revealed that the NAO staining profiles were not different in round and two-cell division intermediates in the uninduced Cls_6xH transformant and in wild-type *Chlamydia trachomatis* serovar L2. The NAO staining profiles in early and mid/late division intermediates were statistically different in the uninduced Cls_6xH transformant and in wild type *Chlamydia trachomatis* (* p is less than or equal to 0.03). This difference was primarily due to an additional category of NAO staining (diffuse in one cell) that was detected in early and mid/late division intermediates in wild-type *Chlamydia trachomatis* and was not detected in the uninduced Cls_6xH transformant. Images in (A and C) were acquired with a Zeiss AxioImager2 microscope equipped with a 100x oil immersion PlanApochromat lens. Scalebar = 2 μm.
(PDF)

S5 Fig Effect of extended overexpression of the Cls_TM_GFP construct on chlamydial morphology. Cells were infected as described in the legend of Fig 3, and expression of Cls_TM_GFP was induced or not at 8hpi with 2 or 10nM aTc. Cells were fixed at 24hpi and processed for immunofluorescence using antibodies against the major outer membrane protein (MOMP). DNA was visualized with DAPI. Images were acquired on a Zeiss AxioImager.Z2 equipped with an Apotome2 using a 100X lens objective. Images are representative of at least three independent experiments. Scalebar = 2 μm.
(PDF)

S6 Fig Effect of diNN on localization of Cls_TM_GFP. Samples were collected and processed as described in the legend to Fig 4. (A) Representative images of diNN-treated Cls_TM_GFP expressing bacteria at each stage of division. (B) 50 individual cells from Early, Mid/late and Two-cell stages of division from untreated and diNN-treated cultures were assessed for the localization of Cls_TM_GFP. Localization profiles were categorized into leading edge of the budding daughter cell, diffuse in mother cell, diffuse in one cell, diffuse in both cells, or discrete in one cell. The differences in Cls_TM_GFP localization between treatment conditions at each stage of division were statistically analyzed using a chi-squared test to reveal that the changes observed during diNN treatment were statistically significant (p<0.001).
(PDF)

S7 Fig Effect of diNN on localization of PsdD_6xH. Samples were collected and processed as described in the legend to Fig 4. Representative images of untreated (UTD) or diNN-treated PsdD_6xH expressing bacteria (labeled PsdD) at each stage of division are shown. 50 individual cells from each stage of division from untreated and 35 cells from diNN-treated cultures were assessed for their localization to the leading edge of the budding daughter cell, the mother cell, or both cells for Early and Mid/Late stages, whereas, for the Two-cell stage, discrete localization in one cell or diffuse localization in one or both cells was quantified. The differences in localization of PsdD_6xH between treatment conditions at each stage of division were statistically analyzed using a chi-squared test to reveal that the changes observed during diNN treatment were not statistically significant.
(PDF)

S8 Fig **(A)** The detection of C11orf83_6xH constructs by western blotting. McCoy cells were infected with the transformants carrying an aTc-inducible plasmid encoding C11orf83_6xH, Cls_TM-c11orf83_6xH, or OppB_TM-c11orf83_6xH. At 16 hpi, the constructs were induced with 2 or 10 nM aTc, and the infected cells were harvested at 24 hpi and processed for western blotting as described in the Materials and Methods. Protein samples were separated by SDS-PAGE, transferred to PVDF, and blotted with mouse anti-6xH (800) and goat anti-MOMP (680). Samples from three independent experiments were analyzed and a representative blot is shown. (B) Expression of the constructs under the conditions used for western blotting was further verified by indirect immunofluorescence assay. The images were acquired on Zeiss AxioImager.Z2 equipped with an Apotome2 using a 100X lens objective. Images are representative of at least three independent experiments. Scalebar = 2 μm. MOMP = major outer membrane protein (green in panel B). 6xH indicates the specific C11orf83 construct indicated (red in panel B) in a merged image for each strain under different induction conditions (0, 2, or 10nM aTc).
(PDF)

S9 Fig Cls_6xH and anionic phospholipid localization is unaffected by disrupting MreB activity. Cells were either pre-treated with A22 for 2h prior (Pre) to inducing (A) Cls_6xH or (B) MreB_6xH expression at 16hpi or treated after inducing (Post) at 18hpi. See legend to Fig 6 for more details. (C) The effect of A22 on the distribution of Cls_6xH, anionic phospholipids, and MreB_6xH. Cells were infected with the Cls_6xH or MreB_6xH *Chlamydia* transformant, or with non-transformed wild-type *C*. *trachomatis* serovar L2. Cells were induced by the addition of 10nM aTc to the media at 20hpi or uninduced as shown in the figure. Infected cells were also incubated in the absence or presence of 75μM A22 for 2hrs prior to lysis at 22hpi. The cells in the lysate were fixed and stained with MOMP and 6xHis antibodies, or NAO as described in the Materials and Methods. Representative images illustrating the localization of Cls_6xH, MreB_6xH, or NAO-staining aPLs are shown for each condition. (D) The distribution of Cls_6xH, MreB_6xH, or NAO-staining aPLs was quantified in round cells from two independent experiments. Localization profiles for Cls_6xH, MreB_6xH, and NAO-staining aPLs were categorized into polar or diffuse in one cell. For D data were pooled from two independent experiments. Chi-squared analysis revealed that A22 treatment did not have a statistically significant effect on the distribution of Cls_6xH and NAO-staining aPLs, but it did affect the distribution of MreB (*** = p < 0.00001).
(PDF)

S10 Fig MreB_6xH localization in dividing *Chlamydia*. Hela cells were infected with the aTc-inducible MreB_6xH transformant, and expression of the construct was induced by the addition of 10nM aTc to the media at 20hpi. Infected cells were lysed at 22hpi and the localization of MreB_6xH (labeled MreB) in the chlamydial cells in the lysate during various stages of division is shown. See also Fig 7B to compare the localization of MreB_6xH induced in chlamydial cells that were incubated in axenic media. Scalebar = 2 μm.
(PDF)
