# Supplementary material for: Localized cardiolipin synthesis is required for the assembly of MreB during the polarized cell division of Chlamydia trachomatis
Source: PLoS Pathog. 2022 Sep 12;18(9):e1010836. doi: 10.1371/journal.ppat.1010836 (PMC9499288; doi:10.1371/journal.ppat.1010836)
Supplement: S1 Table — (PDF) [file ppat.1010836.s001.pdf]

S1 Table. List of Plasmids and Primers

Plasmid	Relevant genotype	Ori	Source or Reference
pBOMB4-Tet::L2	*bla* P*tet*::*mCherry* P_{Nm}::*gfp*	pUC19	1
pBOMB-cls_6xH::L2	*bla* P*tet*::*cls_6xH* P_{Nm}::*gfp*	pUC19	This study
pBOMB-ΔN25_cls_6xH::L2	*bla* P*tet*::ΔN25_*cls_6xH* P_{Nm}::*gfp*	pUC19	This study
pBOMB-psdD_6xH::L2	*bla* P*tet*::*psdD_6xH* P_{Nm}::*gfp*	pUC19	This study
pTLR2-*mreB*_6xH	*bla* P*tet*::*mreB*_6xH $P_{dnaKmut}$::*mKate2*	ColE1	2
pBOMBmC::L2	*bla* P*tet*::*mCherry* P_{Nm}::*mCherry*	pUC19	3
pBOMBmC-cls_TM_GFP::L2	*bla* P*tet*::*cls_TM_GFP* P_{Nm}::*mCherry*	pUC19	This study
pBOMB-c11orf83_6xH::L2	*bla* P*tet*::*c11orf83_6xH* P_{Nm}::*gfp*	pUC19	This study
pBOMB-cls_TM_c11orf83_6xH::L2	*bla* P*tet*::*cls_TM_c11orf83_6xH* P_{Nm}::*gfp*	pUC19	This study
pBOMB-TM_c11orf83_6xH::L2	*bla* P*tet*:: TM_{Ec_OppB}_*c11orf83_6xH* P_{Nm}::*gfp*	pUC19	This study
pBOMB-TM_mCherry::L2	*bla* P*tet*:: TM_{Ec_OppB}_*mCherry* P_{Nm}::*gfp*	pUC19	This study
pSTM25	*aadA* P*lac*:: *t25*-TM_{Ec_OppB}	p15A	4
pSTM25-cls	*aadA* P*lac*:: *t25*-TM_{Ec_OppB}-*cls*	p15A	This study

Primer name	Sequence	Features	Usage
cls/(pBOMB)/5'	gatctaaagaggagaaaggatctgcATGAAAATGGCTTTTTACG	lower case for plasmid overlap construction	For amplification of cls into pBOMB
cls_6xH/(pBOMB)/3'	tttgaatggtcgaccggtacctgcattaatggtgatggtgatggtgAGATGGCATGTATCTCTGTTC	lower case for plasmid overlap construction; adds 6xH sequence to cls	For amplification of cls into pBOMB
psdD/(pBOMB)/5'	gatctaaagaggagaaaggatctgcATGGCAGCGCGGGAAATG	lower case for plasmid overlap construction	For amplification of psdD into pBOMB
psdD_6xH/(pBOMB)/3'	tttgaatggtcgaccggtacctgcattaatggtgatggtgatggtgTGAAGAGAAACGTTTTCCTAACGATTG	lower case for plasmid overlap construction; adds 6xH sequence to cls	For amplification of psdD into pBOMB
cls/(pSTM25)/5’	acgcacaagggcctctagagATAGCTATTCCGGATGGAG	lower case for plasmid overlap construction	For amplification of cls into pSTM25

cls/(pSTM25)/3’	attcttagttacttaggtacttaAGATGGCATGTATCTC	lower case for plasmid overlap construction	For amplification of cls into pSTM25
N26-cls/(pBOMB)/5’	aaagaggagaaaggatctgcATGGCTATTCCGGATGGAGAC	lower case for plasmid overlap construction	For amplification of ΔN25_cls into pBOMB
cls_TM/(pBOMBmC)/5’	aaagaggagaaaggatctgcATGAAAATGGCTTTTTTACGG	lower case for plasmid overlap construction	For amplification of cls_TM fragment
cls_TM/(GFP)/3’	ctcctttactAGTGTGCGCAAAACCATTC	lower case for overlap with GFP	For amplification of cls_TM fragment
GFP/(cls_TM)/5’	tgcgcacactAGTAAAGGAGAAGCACTTTTC	lower case for overlap with cls_TM	For amplification of GFP
GFP/(pBOMB)/3’	tttgaatggtcgaccggtacTTATTTGTATAGTTCATCCATGCCATG	lower case for plasmid overlap construction	For amplification of GFP
ct284 cls qPCR F	CACCTGTTGGCCGCTATTA		Transcript analysis
ct284 cls qPCR R	GCGCTGAAGAACTGTGGATA		Transcript analysis
ct446 euo qPCR F	CGAAGACTACTCGTTGGGAAATA		Genomic DNA quantification
ct446 euo qPCR R	AACAGAAGCTCTCCTTGATAAGT		Genomic DNA quantification

gBlock name	Sequence	Features	Usage
c11orf83_6xH (pBOMB)	aaagaggagaaaggatctgcATGGACTCACTCCGTAAGATGTTAATATCCGTCGCTATGCTTGGAGCTGGTGCAGGTGTTGGCTACGCATTACTAGTTATCGTGACCCCCGGCGAACGCAGAAAACAGGAAATGCTCAAGGAAATGCCTTTACAGGACCCACGTTCAAGAGAAGAAGCGGCCCGAACGCAACAGTTACTCTTAGCAACCTTACAGGAAGCTGCTACAACACAGGAGAATGTTGCCTGGAGAAAAAATTGGATGGTAGGTGGGGAAGGCGGTGCAGGTGGAAGATCCCCG**CACCATCACCATCACCAT**TAAgtaccggtcgaccattcaaa	lower case for plasmid overlap, bolded is 6xH	Insert codon-optimized c11orf83_6xH into pBOMB
cls_TM_c11orf83_6xH (pBOMB)	aaagaggagaaaggatctgcATGAAAATGGCTTTTTTACGGAAAATATTTGTATTTGTAGCTTGTGTTGTCTCGTTGAATGGTTTTGCGCACACTGACTCACTCCGTAAGATGTTAATATCCGTCG	lower case for plasmid overlap, underlined sequence is cls_TM, bolded is 6xH	Insert codon-optimized cls_TM_c11orf83_6x

	CTATGCTTGGAGCTGGTGCAGGTGTTGGCTACGCATTACTAGTTATCGTGACCCCCGGCGAACGCAGAAAACAGGAAATGCTCAAGGAAATGCCTTTACAGGACCCACGTTCAAGAGAAGAAGCGGCCCGAACGCAACAGTTACTCTTAGCAACCTTACAGGAAGCTGCTACAACACAGGAGAATGTTGCCTGGAGAAAAAATTGGATGGTAGGTGGGGAAGGCGGTGCAGGTGGAAGATCCCCG**CACCATCACCATCACCAT**TAAgtaccggtcgaccattcaaa		H into pBOMB
TM_c11orf83_6xH (pBOMB)	gatctaaagaggagaaaggatctgcATGTTAAAATTTATTCTACGTCGCTGTCTGGAAGCGATTCCGACGCTATTTATTCTTATTACTATTTCGTTCTTTATGATGCGCCTCGCGCCGGGAAGCCCTTTTACC*GGTGGATCA*TTACTAGTTATCGTGACCCCCGGCGAACGCAGAAAACAGGAAATGCTCAAGGAAATGCCTTTACAGGACCCACGTTCAAGAGAAGAAGCGGCCCGAACGCAACAGTTACTCTTAGCAACCTTACAGGAAGCTGCTACAACACAGGAGAATGTTGCCTGGAGAAAAAATTGGATGGTAGGTGGGGAAGGCGGTGCAGGTGGAAGATCCCCG**CACCATCACCATCACCAT**TAAgtaccggtcgaccattcaaatatgt	lower case for plasmid overlap, underlined sequence is oppB TM from *E. coli*, italicized sequence is GGS linker, bolded is 6xH, c11orf83 sequence lacks any predicted membrane-targeting motifs (ΔN23)	Insert codon-optimized TM_c11orf83_6xH into pBOMB
Ec_oppB_TM (pBOMB)	gatctaaagaggagaaaggatctgcATGTTAAAATTTATTCTACGTCGCTGTCTGGAAGCGATTCCGACGCTATTTATTCTTATTACTATTTCGTTCTTTATGATGCGCCTCGCGCCGGGAAGCCCTTTTACC*GGTGGATCA*ggcatggtctctaagggcgaggaaga	lower case for plasmid overlap, underlined sequence is oppB TM from *E. coli*, italicized sequence is GGS linker	Insert *E. coli* OppB 1st TM domain into pBOMB

1 Bauler, L. D. & Hackstadt, T. Expression and targeting of secreted proteins from Chlamydia trachomatis. *J Bacteriol* **196**, 1325-1334, doi:10.1128/JB.01290-13 (2014).

2. Lee, J., Cox, J.V., & Ouellette, S. P. Critical role for the extended N-terminus of chlamydial MreB in directing its membrane association and potential interaction with divisome proteins. *J Bacteriol* **202**, e00034-20 (2020).

3. Wood, N. A., Blocker, A. M., Seleem, M. A., Conda-Sheridan, M., Fisher, D. J., & Ouellette, S. P. The ClpX and ClpP2 orthologs of *Chlamydia trachomatis* perform discrete and essential functions in organism growth and development. *mBio* **11**, e02016-20 (2020).

4 Ouellette, S. P., Gauliard, E., Antosova, Z, & Ladant, D. A Gateway®-compatible bacterial adenylate cyclase-based two-hybrid system. *Environ Microbiol Rep* **6**: 259-267 (2014).
