# Supplementary figures and images for: Localized cardiolipin synthesis is required for the assembly of MreB during the polarized cell division of Chlamydia trachomatis
Source: PLoS Pathog. 2022 Sep 12;18(9):e1010836. doi: 10.1371/journal.ppat.1010836 (PMC9499288; doi:10.1371/journal.ppat.1010836)

**A**

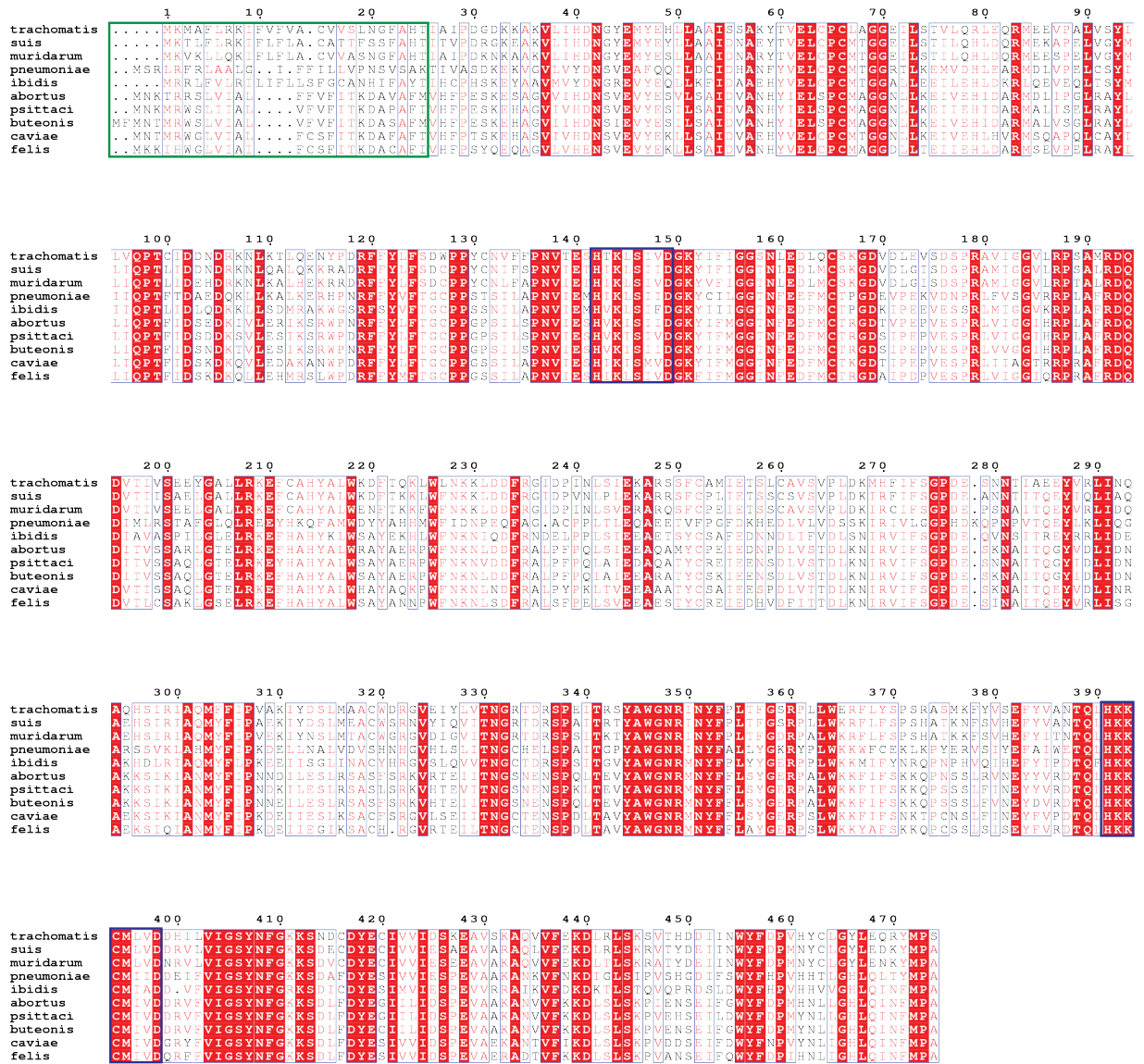

**B**

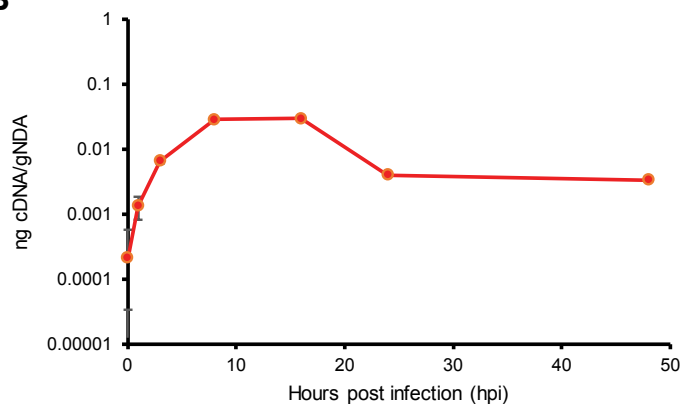

**S1 Fig**

Supplement: S1 Fig — (A) Alignment of ct284/cls orthologs from multiple pathogenic chlamydial species. The numbering is for the C. trachomatis protein. The predicted transmembrane domain is boxed in green with the catalytic sites boxed in black. White lettering on red background indicates conservation of the residues across all species whereas red lettering on white background indicates similarity at that position. (B) Transcriptional analysis of cls during the C. trachomatis L2 developmental cycle. Cells were infected with wild-type C. trachomatis L2/434Bu, and total RNA and DNA were collected at the indicated time points. Transcript levels were normalized to genomic DNA levels and are expressed as ng cDNA/gDNA. Shown are data from one of two experiments. (PDF) [file ppat.1010836.s002.pdf]

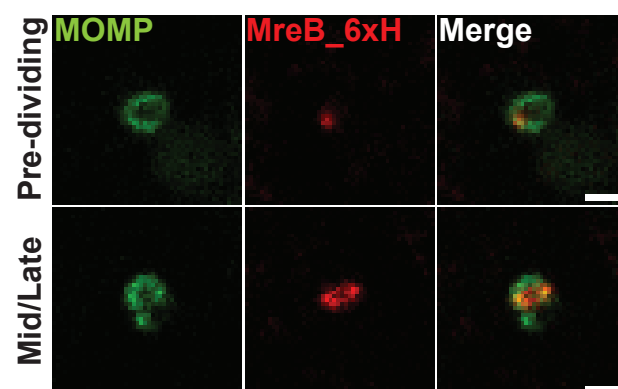

S2 Fig

Supplement: S2 Fig — HeLa cells were infected with the transformant carrying an aTc-inducible plasmid encoding MreB_6xH. At 4 hpi, the construct was induced with 10 nM aTc, and the cells were fixed at 10.5 hpi. Images were acquired on a Nikon Ti2 spinning disc confocal microscope using a 60X lens objective. Images are representative of at least three independent experiments. Scalebar = 2 μm. (PDF) [file ppat.1010836.s003.pdf]

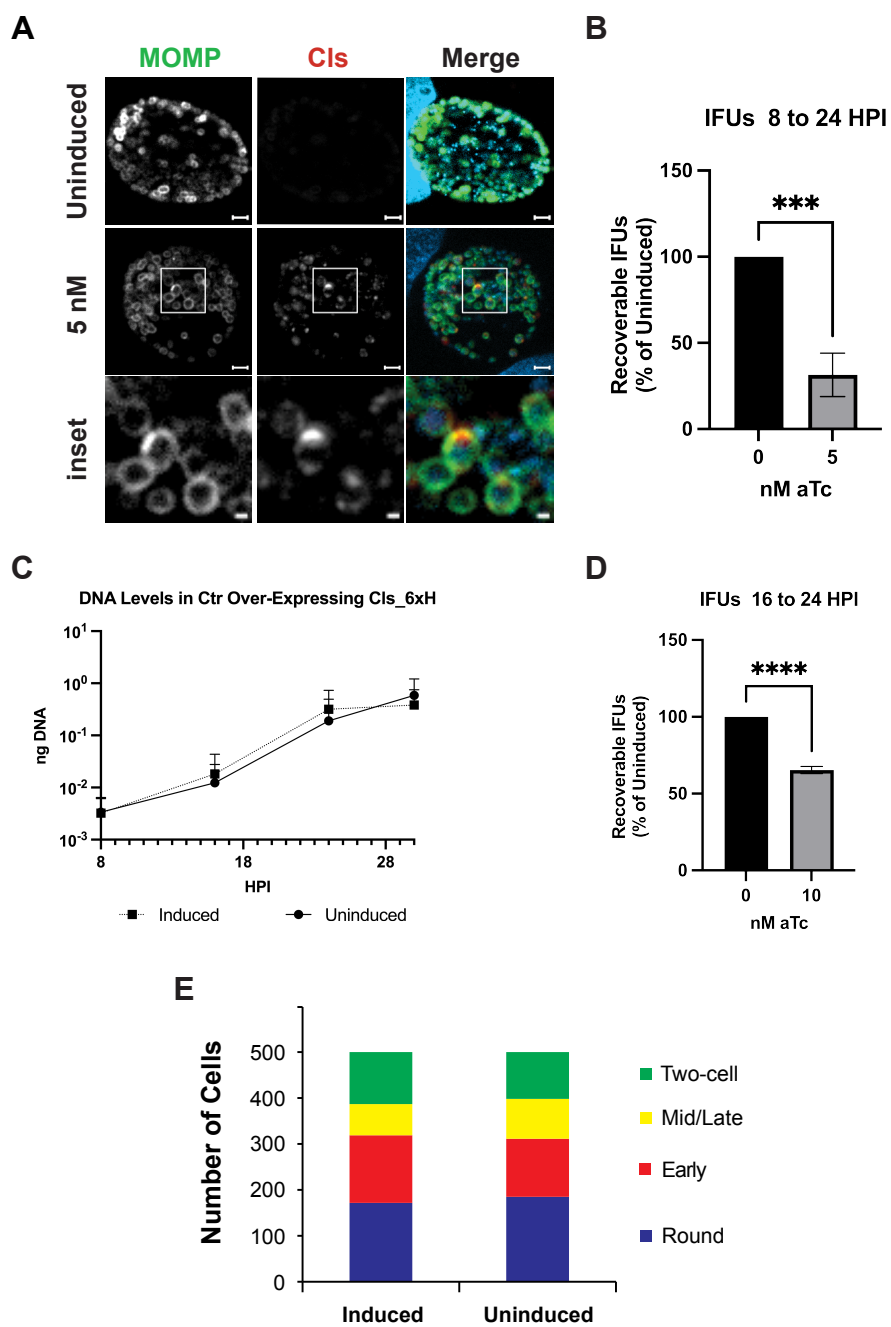

S3 Fig

Supplement: S3 Fig — (A) Localization of wild-type Cls_6xH (labeled Cls) in C. trachomatis L2. Cells were infected, induced or not for expression at 4hpi with 5nM anhydrotetracycline (aTc), and samples were collected and processed at 24hpi. Bacteria were labeled with an antibody targeting MOMP (major outer membrane protein—green), and the construct was labeled with an antibody against the 6xH tag (red). Nuclear DNA was labeled with DAPI and is visualized within the merged image only in the blue channel. The boxed region within each induced image is shown below as an enlarged inset. Images were acquired on a Zeiss AxioImager.Z2 equipped with an Apotome2 using a 100X lens objective. Images are representative of at least three independent experiments. Scalebar of full inclusion images = 2 μm. Scalebar of inset = 0.5 μm. (B) Cells were infected with Cls_6xH transformant and processed as described in the Materials and Methods to quantify IFU production during the primary infection. The uninduced values were arbitrarily set to 100%, and the effect of overexpression at 5nM aTc when added at 8hpi is expressed as a percentage of the wild type. Data are the average of at least three biological replicates assayed in triplicate. *** = p = 0.0007. (C) Quantification of genomic DNA over a time course of infection for the Cls_6xH transformant in uninduced and induced (5nM aTc) conditions. Cell lysates were collected at the indicated time points, and genomic DNA was extracted and quantified by qPCR as described in Materials and Methods. No statistical differences were observed between the uninduced and induced conditions for any of the time points assessed. Data are the average of three independent experiments assayed in triplicate. (D) Cells were infected with Cls_6xH transformant, expression was induced or not at 16hpi with 10nM aTc, and IFUs were quantified at 24hpi. The uninduced values were arbitrarily set to 100%, and the effect of overexpression at 10nM aTc when added at 16hpi is expressed as a [file ppat.1010836.s004.pdf]

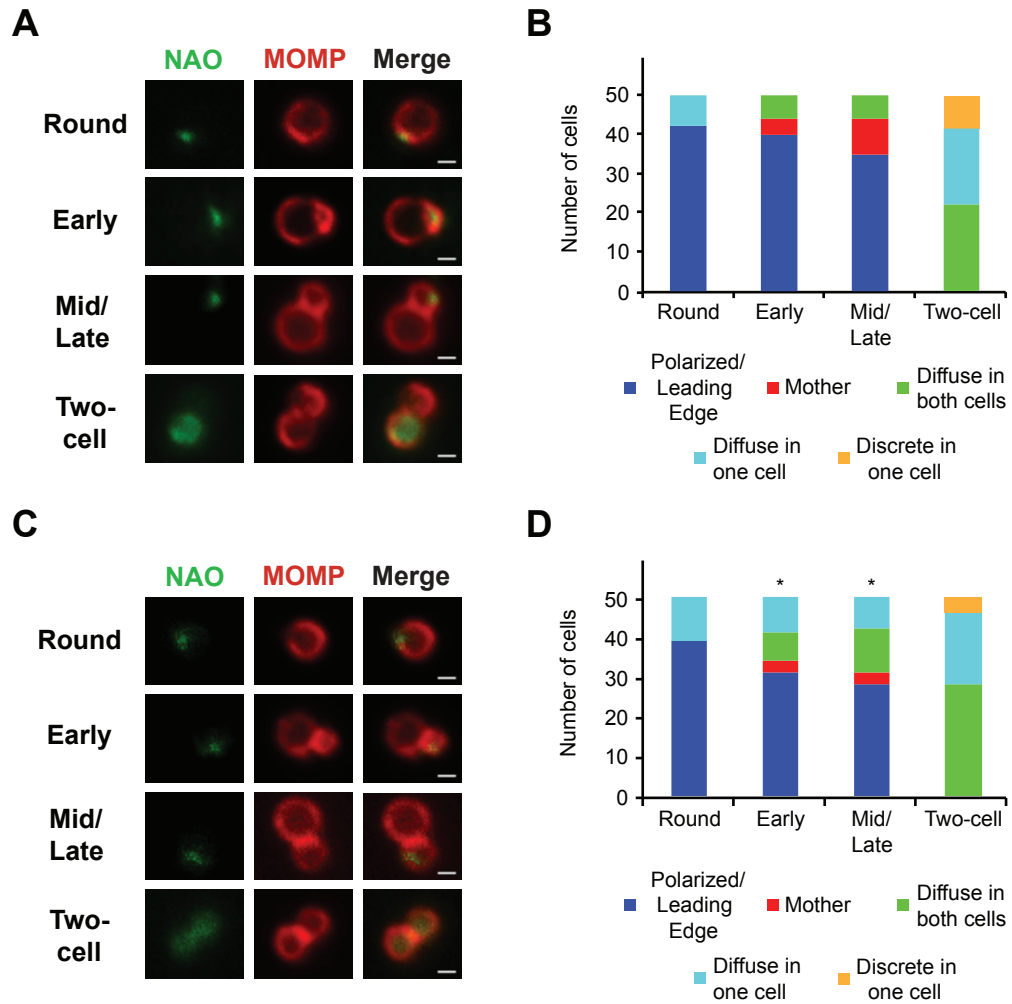

S4 Fig

Supplement: S4 Fig — HeLa cells were infected with the Cls_6xH transformant that was not induced (A and B) or with wild-type C. trachomatis serovar L2 (C and D). At 22hpi, Chlamydia were released from infected cells and the distribution of (A and C) MOMP and endogenous aPLs (stained with 250nM NAO for 2 hours) within chlamydial cells in the lysate was assessed. Representative images illustrating the localization profiles for each marker in Round cells (pre-division intermediates), and in the Early, Mid/Late, and Two-cell stages of the polarized division process are shown. The distribution of (B and D) MOMP and endogenous aPLs was evaluated in 50 individual cells from the indicated stages of division. Localization profiles for endogenous NAO-staining phospholipids were categorized into leading edge of the budding daughter cell/polar, diffuse in mother cell, diffuse in one cell, diffuse in both cells. Chi-squared analysis revealed that the localization profiles of endogenous aPLs in the uninduced Cls_6xH transformant (B) were not statistically different than the localization profiles of aPLs in cells where Cls_6xH expression was induced by the addition of 10nM aTc to the media of infected cells (Fig 2C). Chi-squared analysis revealed that the NAO staining profiles were not different in round and two-cell division intermediates in the uninduced Cls_6xH transformant and in wild-type Chlamydia trachomatis serovar L2. The NAO staining profiles in early and mid/late division intermediates were statistically different in the uninduced Cls_6xH transformant and in wild type Chlamydia trachomatis (* p is less than or equal to 0.03). This difference was primarily due to an additional category of NAO staining (diffuse in one cell) that was detected in early and mid/late division intermediates in wild-type Chlamydia trachomatis and was not detected in the uninduced Cls_6xH transformant. Images in (A and C) were acquired with a Zeiss AxioImager2 microscope equipped with a 100x oil immersion PlanApochr [file ppat.1010836.s005.pdf]

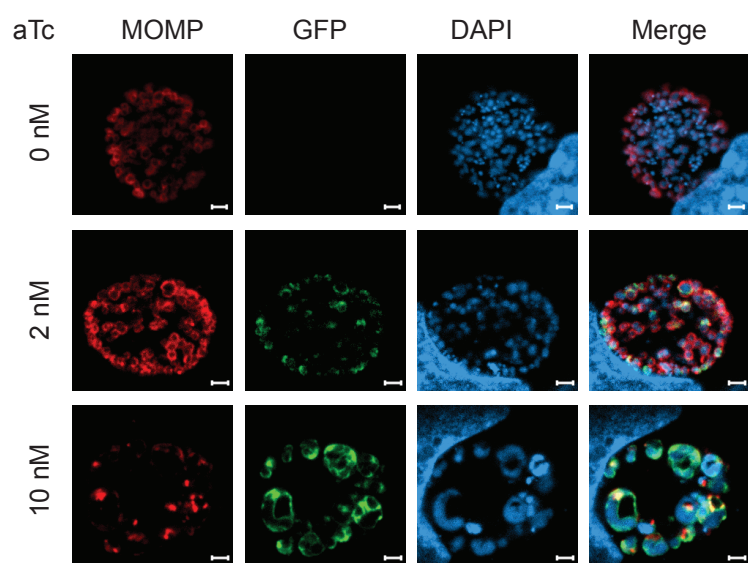

**S5 Fig**

Supplement: S5 Fig — Cells were infected as described in the legend of Fig 3, and expression of Cls_TM_GFP was induced or not at 8hpi with 2 or 10nM aTc. Cells were fixed at 24hpi and processed for immunofluorescence using antibodies against the major outer membrane protein (MOMP). DNA was visualized with DAPI. Images were acquired on a Zeiss AxioImager.Z2 equipped with an Apotome2 using a 100X lens objective. Images are representative of at least three independent experiments. Scalebar = 2 μm. (PDF) [file ppat.1010836.s006.pdf]

**A**

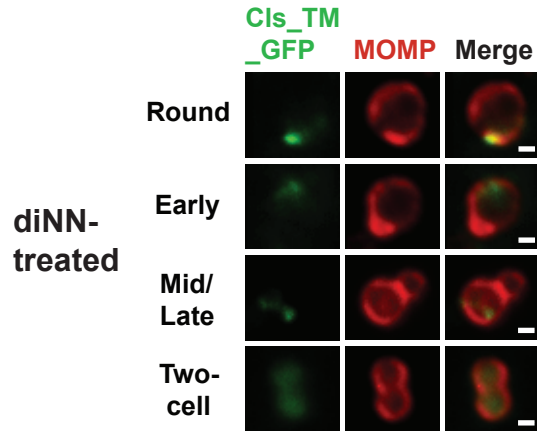

**B**

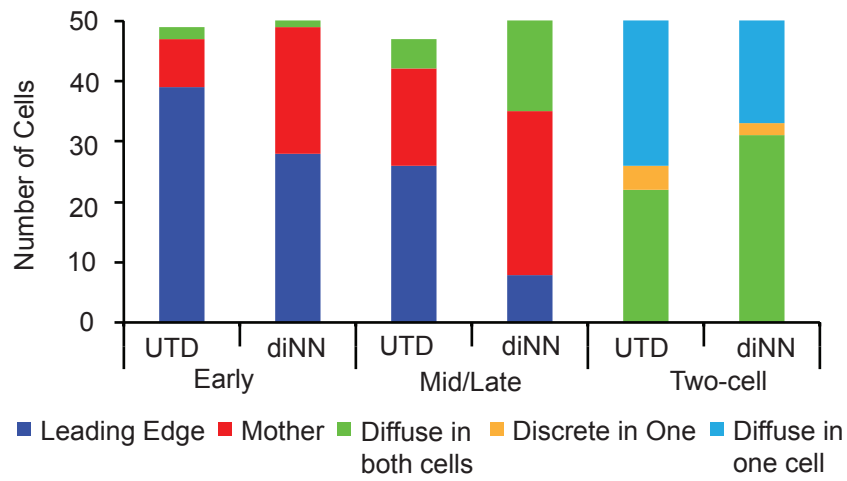

**S6 Fig**

Supplement: S6 Fig — Samples were collected and processed as described in the legend to Fig 4. (A) Representative images of diNN-treated Cls_TM_GFP expressing bacteria at each stage of division. (B) 50 individual cells from Early, Mid/late and Two-cell stages of division from untreated and diNN-treated cultures were assessed for the localization of Cls_TM_GFP. Localization profiles were categorized into leading edge of the budding daughter cell, diffuse in mother cell, diffuse in one cell, diffuse in both cells, or discrete in one cell. The differences in Cls_TM_GFP localization between treatment conditions at each stage of division were statistically analyzed using a chi-squared test to reveal that the changes observed during diNN treatment were statistically significant (p<0.001). (PDF) [file ppat.1010836.s007.pdf]

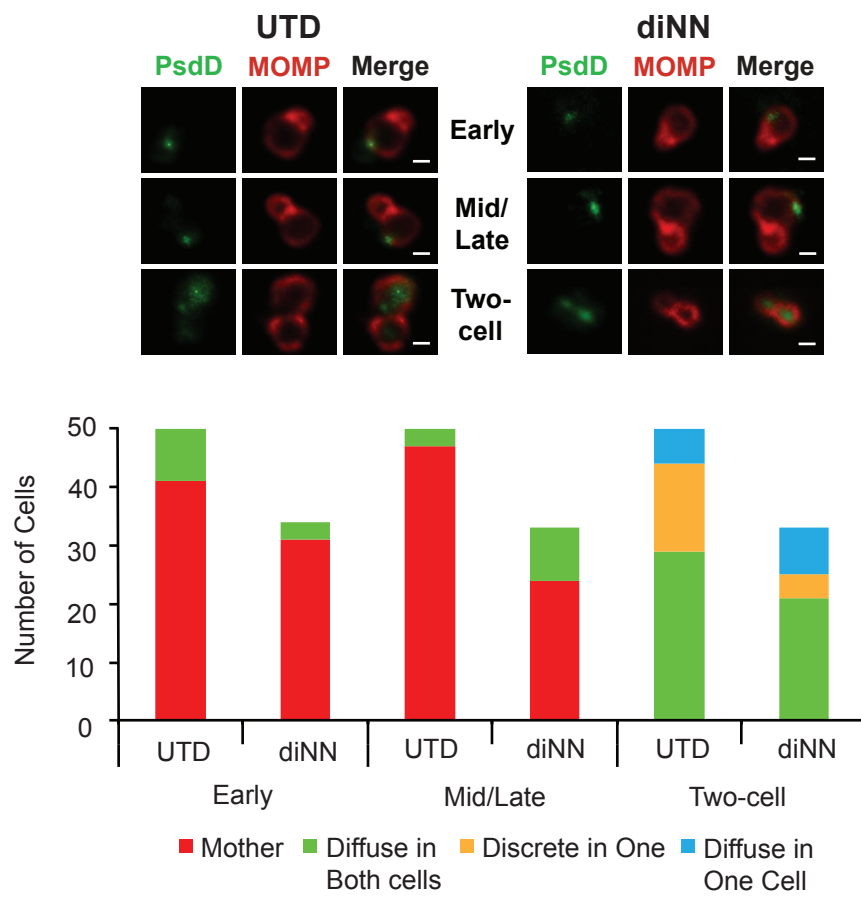

**S7 Fig**

Supplement: S7 Fig — Samples were collected and processed as described in the legend to Fig 4. Representative images of untreated (UTD) or diNN-treated PsdD_6xH expressing bacteria (labeled PsdD) at each stage of division are shown. 50 individual cells from each stage of division from untreated and 35 cells from diNN-treated cultures were assessed for their localization to the leading edge of the budding daughter cell, the mother cell, or both cells for Early and Mid/Late stages, whereas, for the Two-cell stage, discrete localization in one cell or diffuse localization in one or both cells was quantified. The differences in localization of PsdD_6xH between treatment conditions at each stage of division were statistically analyzed using a chi-squared test to reveal that the changes observed during diNN treatment were not statistically significant. (PDF) [file ppat.1010836.s008.pdf]

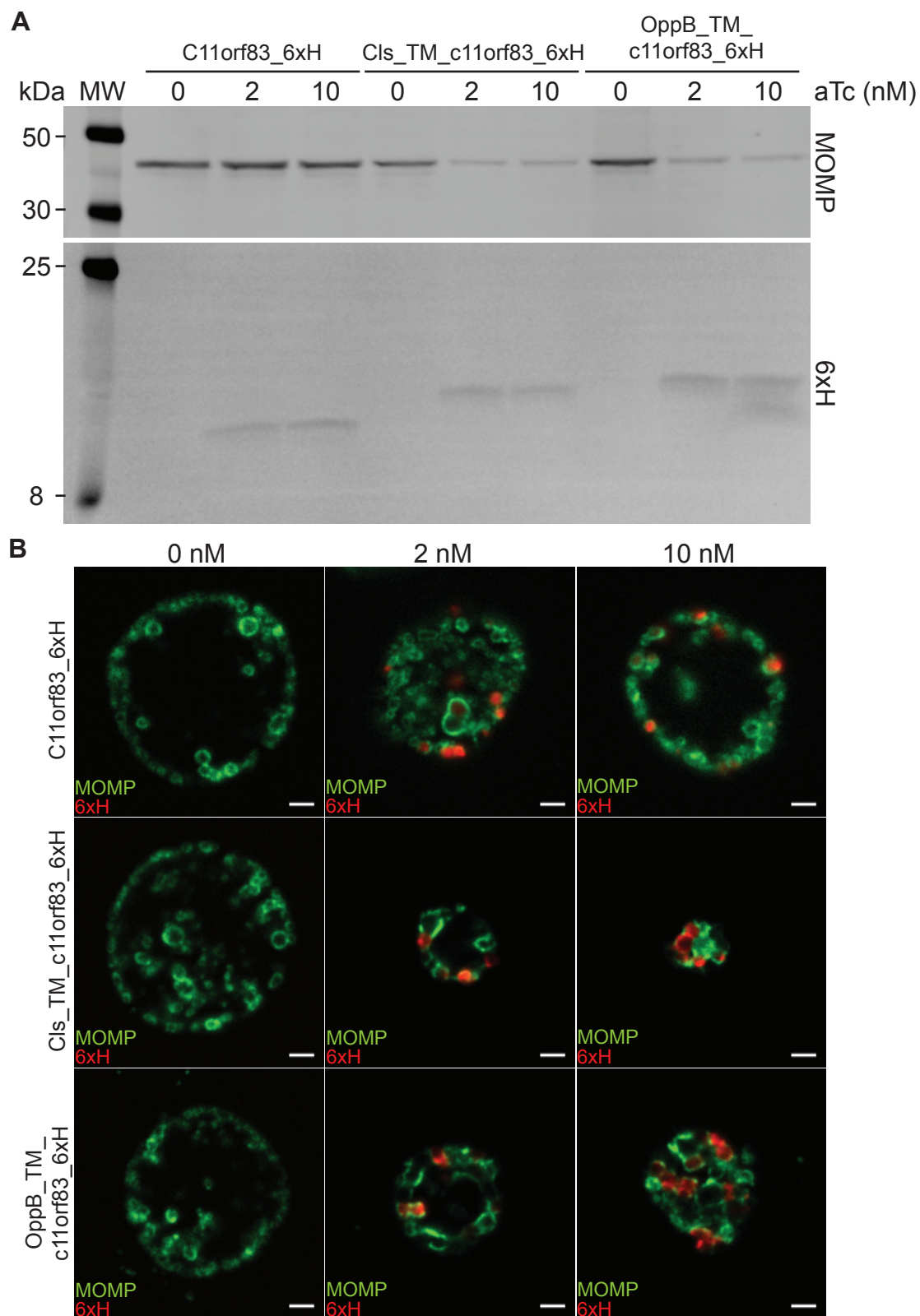

S8 Fig

Supplement: S8 Fig — (A) The detection of C11orf83_6xH constructs by western blotting. McCoy cells were infected with the transformants carrying an aTc-inducible plasmid encoding C11orf83_6xH, Cls_TM-c11orf83_6xH, or OppB_TM-c11orf83_6xH. At 16 hpi, the constructs were induced with 2 or 10 nM aTc, and the infected cells were harvested at 24 hpi and processed for western blotting as described in the Materials and Methods. Protein samples were separated by SDS-PAGE, transferred to PVDF, and blotted with mouse anti-6xH (800) and goat anti-MOMP (680). Samples from three independent experiments were analyzed and a representative blot is shown. (B) Expression of the constructs under the conditions used for western blotting was further verified by indirect immunofluorescence assay. The images were acquired on Zeiss AxioImager.Z2 equipped with an Apotome2 using a 100X lens objective. Images are representative of at least three independent experiments. Scalebar = 2 μm. MOMP = major outer membrane protein (green in panel B). 6xH indicates the specific C11orf83 construct indicated (red in panel B) in a merged image for each strain under different induction conditions (0, 2, or 10nM aTc). (PDF) [file ppat.1010836.s009.pdf]

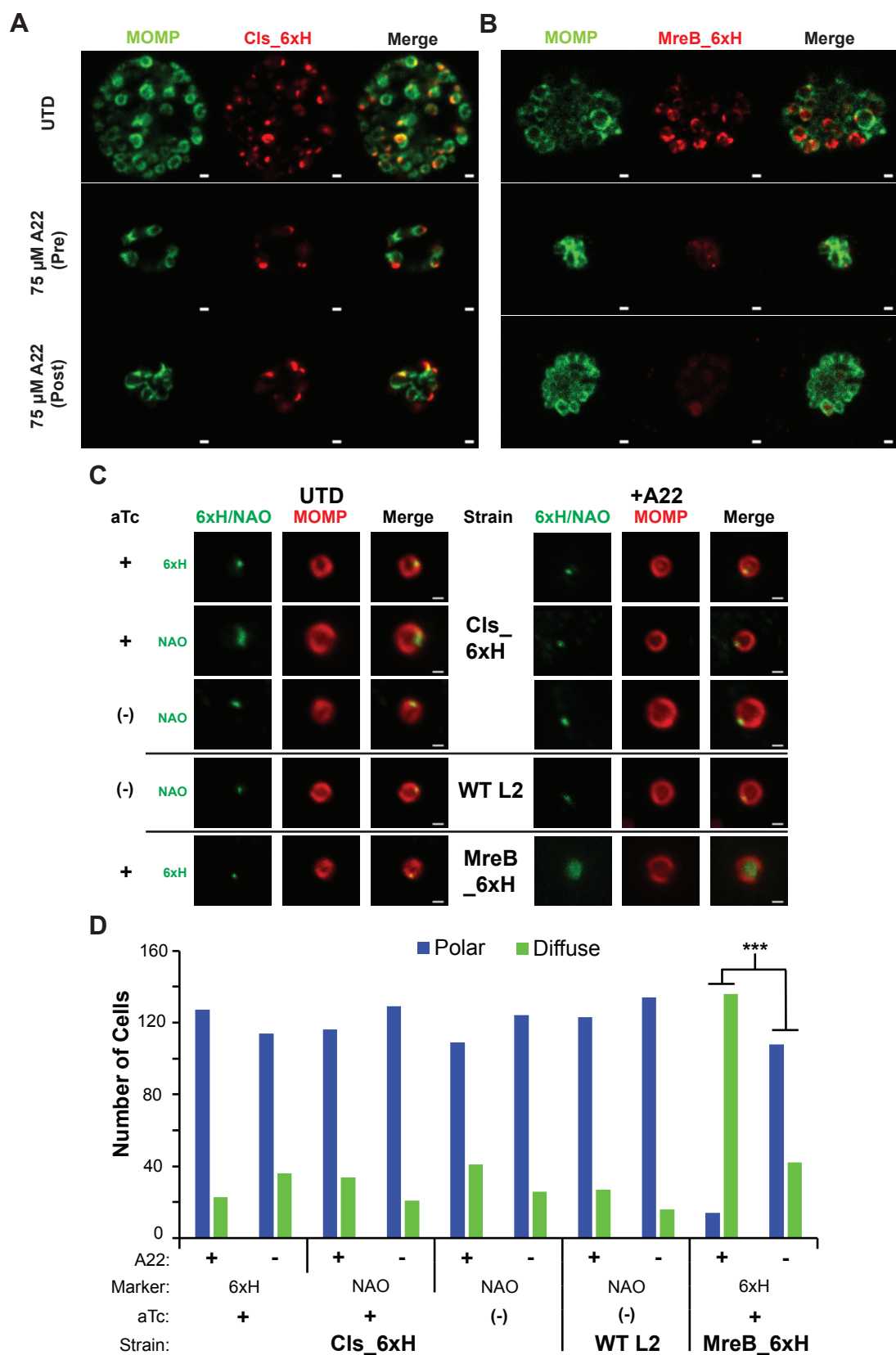

S9 Fig

Supplement: S9 Fig — Cells were either pre-treated with A22 for 2h prior (Pre) to inducing (A) Cls_6xH or (B) MreB_6xH expression at 16hpi or treated after inducing (Post) at 18hpi. See legend to Fig 6 for more details. (C) The effect of A22 on the distribution of Cls_6xH, anionic phospholipids, and MreB_6xH. Cells were infected with the Cls_6xH or MreB_6xH Chlamydia transformant, or with non-transformed wild-type C. trachomatis serovar L2. Cells were induced by the addition of 10nM aTc to the media at 20hpi or uninduced as shown in the figure. Infected cells were also incubated in the absence or presence of 75μM A22 for 2hrs prior to lysis at 22hpi. The cells in the lysate were fixed and stained with MOMP and 6xHis antibodies, or NAO as described in the Materials and Methods. Representative images illustrating the localization of Cls_6xH, MreB_6xH, or NAO-staining aPLs are shown for each condition. (D) The distribution of Cls_6xH, MreB_6xH, or NAO-staining aPLs was quantified in round cells from two independent experiments. Localization profiles for Cls_6xH, MreB_6xH, and NAO-staining aPLs were categorized into polar or diffuse in one cell. For D data were pooled from two independent experiments. Chi-squared analysis revealed that A22 treatment did not have a statistically significant effect on the distribution of Cls_6xH and NAO-staining aPLs, but it did affect the distribution of MreB (*** = p < 0.00001). (PDF) [file ppat.1010836.s010.pdf]

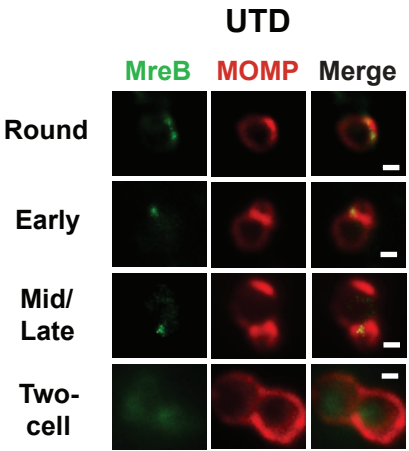

**S10 Fig**

Supplement: S10 Fig — Hela cells were infected with the aTc-inducible MreB_6xH transformant, and expression of the construct was induced by the addition of 10nM aTc to the media at 20hpi. Infected cells were lysed at 22hpi and the localization of MreB_6xH (labeled MreB) in the chlamydial cells in the lysate during various stages of division is shown. See also Fig 7B to compare the localization of MreB_6xH induced in chlamydial cells that were incubated in axenic media. Scalebar = 2 μm. (PDF) [file ppat.1010836.s011.pdf]
